# CRISPR/Cas9-mediated targeted chromosome elimination

**DOI:** 10.1186/s13059-017-1354-4

**Published:** 2017-11-24

**Authors:** Erwei Zuo, Xiaona Huo, Xuan Yao, Xinde Hu, Yidi Sun, Jianhang Yin, Bingbing He, Xing Wang, Linyu Shi, Jie Ping, Yu Wei, Wenqin Ying, Wei Wei, Wenjia Liu, Cheng Tang, Yixue Li, Jiazhi Hu, Hui Yang

**Affiliations:** 1 Institute of Neuroscience, State Key Laboratory of Neuroscience, Key Laboratory of Primate Neurobiology, CAS Center for Excellence in Brain Science and Intelligence Technology, Shanghai Institutes for Biological Sciences, Chinese Academy of Sciences, Shanghai, 200031 China; 2 State Key Laboratory of Membrane Biology and Minstry of Education Key Laboratory of Cell Proliferation and Differentiation, School of Life Sciences, Peking-Tsinghua Center for Life Sciences, Peking University, Beijing, 100871 China; 3 Key Lab of Computational Biology, CAS-MPG Partner Institute for Computational Biology, Shanghai Institutes for Biological Sciences, Chinese Academy of Sciences, Shanghai, 200031 China; 4 University of Chinese Academy of Sciences, Shanghai, 200031 China; 5 Center for Quantitative Sciences, Vanderbilt University School of Medicine, Nashville, Tennessee 37232 USA; 6 Shanghai University, Shanghai, 200444 China; 7 College of Animal Science and Technology, Guangxi University, Nanning, Guangxi 530004 China

## Abstract

**Background:**

The CRISPR/Cas9 system has become an efficient gene editing method for generating cells carrying precise gene mutations, including the rearrangement and deletion of chromosomal segments. However, whether an entire chromosome could be eliminated by this technology is still unknown.

**Results:**

Here we demonstrate the use of the CRISPR/Cas9 system to eliminate targeted chromosomes. Using either multiple cleavages induced by a single-guide RNA (sgRNA) that targets multiple chromosome-specific sites or a cocktail of multiple sgRNAs, each targeting one specific site, we found that a sex chromosome could be selectively eliminated in cultured cells, embryos, and tissues in vivo. Furthermore, this approach was able to produce a targeted autosome loss in aneuploid mouse embryonic stem cells with an extra human chromosome and human induced pluripotent stem cells with trisomy 21, as well as cancer cells.

**Conclusions:**

CRISPR/Cas9-mediated targeted chromosome elimination offers a new approach to develop animal models with chromosome deletions, and a potential therapeutic strategy for human aneuploidy diseases involving additional chromosomes.

**Electronic supplementary material:**

The online version of this article (doi:10.1186/s13059-017-1354-4) contains supplementary material, which is available to authorized users.

## Background

Aneuploidy is a human genetic disorder due to the addition or deletion of a chromosome, leading to significant morbidity and mortality during infancy or childhood [1]. The past decade has witnessed major advances in strategies to correct single-gene defects of rare monogenic disorders, beginning with in vitro experiments and in several cases advancing to in vivo studies and clinical trials. By contrast, only a few attempts have been made to genetically correct the over-dose of genes for an entire chromosome in aneuploid cells. Targeted chromosome elimination could be achieved by insertion of oppositely oriented *loxP* sites into the targeted chromosome followed by Cre-mediated sister-chromatid recombination [2], or by insertion of a *TKNEO* transgene into one copy of a targeted chromosome followed by drug selection of chromosome-deletion clones via spontaneous chromosome loss [3]. Both of these approaches require two-step manipulation and resulted in low yields of chromosome-deleted cells, and are thus unsuitable for in vivo studies. Alternatively, over-dose of genes in aneuploid cells could be corrected by insertion of a large, inducible XIST transgene into the targeted chromosome to silence one copy of it [4]. However, the efficiency of the targeted insertion was very low and some genes may have escaped from inactivation.

The type II bacterial CRISPR/Cas9 system has been engineered into an efficient genome-editing tool consisting of the Cas9 nuclease and a single guide RNA (sgRNA), dramatically transforming our ability to edit the genomes of diverse organisms. The sgRNA targets Cas9 to genomic regions to induce double-stranded DNA breaks, which are repaired by nonhomologous end-joining or homology-directed repair. CRISPR/Cas9-mediated genome editing has been applied to generate cells or animals carrying precise gene mutations [5, 6], including rearrangements [7, 8] and deletion of chromosome segments [9]. We asked whether this powerful technology could be used for targeted chromosome elimination to generate animal models with chromosome deletion in various species and to treat human aneuploidy diseases involving chromosome addition.

In this study we report a novel application of CRISPR/Cas9 technology; the selective elimination of a single specific chromosome via multiple DNA cleavages on the targeted chromosome in cultured cells, embryos, and in vivo tissues. These cleavages were induced by a single sgRNA or two sgRNAs that targeted multiple chromosome-specific sites, or by a cocktail of 14 sgRNAs, with each targeting one specific site. More importantly, this approach eliminated human chromosome 21 (hChr21) in human induced pluripotent stem cells (iPSCs) with trisomy 21. CRISPR/Cas9-mediated targeted chromosome elimination offers a new approach to developing animal models and therapeutic treatments for aneuploidy.

## Results

### Elimination of the Y chromosome in vitro and in vivo

**Fig. 1:**
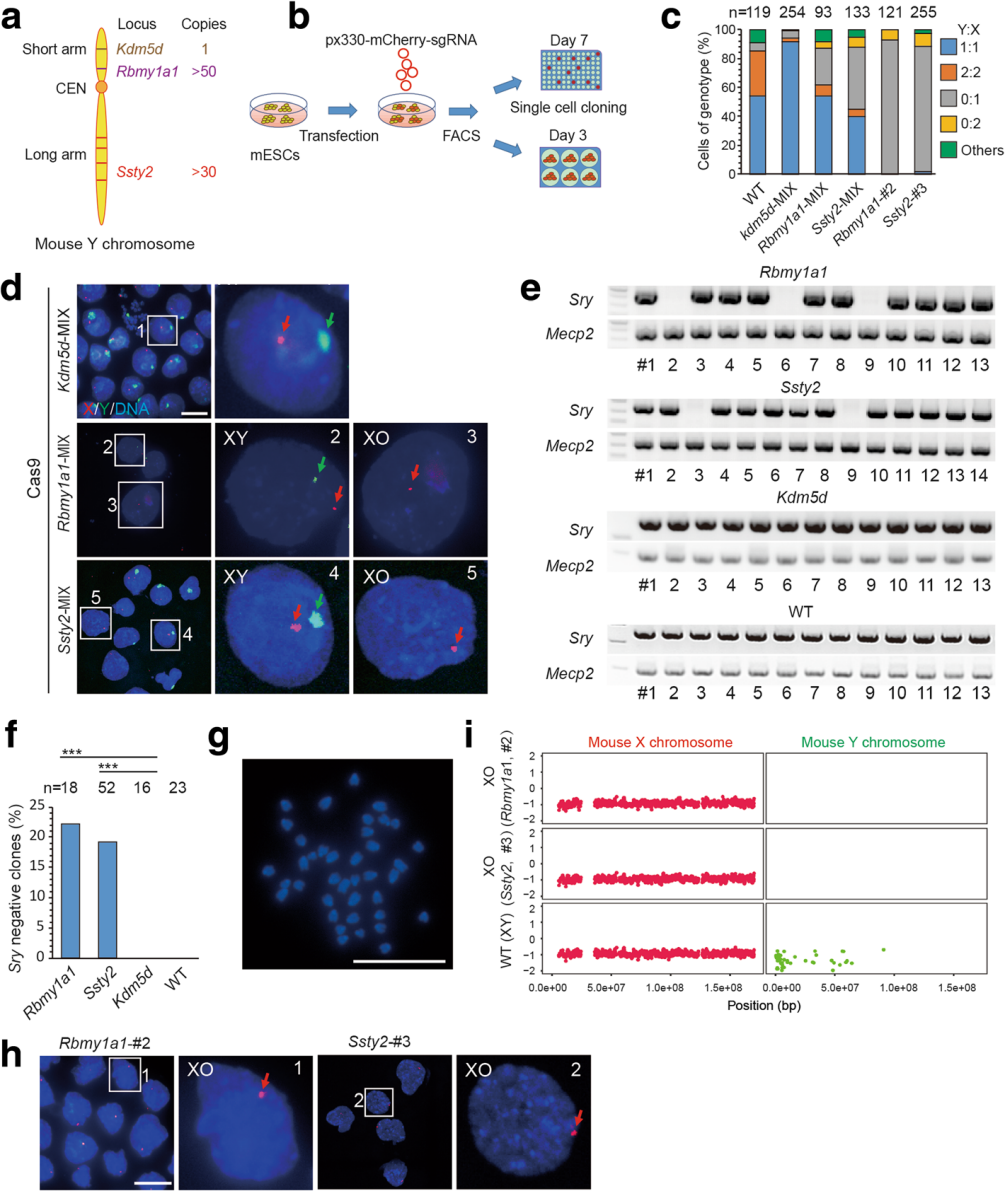
CRISPR/Cas9-mediated Y chromosome elimination in vitro. **a** Targeted gene loci in the Y chromosome: *Rbmy1a1*, clustered in the short arm; *Ssty2*,scattered in the long arm; *Kdm5d*, control gene. **b** Experimental design. Mouse embryonic stem cells (*mESCs*) of XY genotype were transfected with plasmids expressing Cas9, Y chromosome-targeting sgRNAs, and mCherry. One day later, mCherry-positive mESCs were sorted by FACS and cultured in six wells for DNA-FISH analysis or 96 wells for single-cell cloning and genotyping. **c** Stacked bar graphs showing results of DNA-FISH analysis on the gene-edited mESCs. Percentages of cells (including dividing cell, Y:X = 2:2 or 0:2) exhibiting different genotype ratios. *WT* are wild-type, untransfected cells; *n* is the sample size of counted cells. **d** Representative DNA-FISH analysis of mixed ESCs targeted at *Rbmy1a1*, *Ssty2*, or *Kdm5d. Green*, FITC-labeled whole-chromosome probe for Y chromosome; *red*, Texas red-labeled X chromosome probe for XqA7.3; *blue*, Hoechst 33342-labeled DNA. *Green arrows* indicate Y; *red arrows* indicate X. *Numbered squares* indicate single cells shown at a higher resolution in the *right panels. Bar*, 20 μm. **e** Genotyping analysis of ESC clones from *Rbmy1a1* and *Ssty2* targeting. Controls: *Kdm5d* targetted and untransfected (*WT*) cells. *Sry* and *Mecp2* are located on Y and X, respectively. **f** Efficiency of Y chromosome elimination by *Rbmy1a1* and *Ssty2* targeting. The experimental groups (*Rbmy1a1* and *Ssty2*) showed more *Sry*-negative clones than the control group (*Kdm5d* and WT; ***P < 0.001, **P < 0.01, *P < 0.05, Chi-square test). **g** Karyotyping of *Sry*-negative ESCs showed 39 instead of 40 chromosomes. *Bar*, 20 μm. **h** Representative DNA-FISH analysis of single ESC clones targeted at either *Rbmy1a1* or *Ssty2. Bar*, 20 μm. **i** Whole genome sequencing showing Y chromosome elimination of XO ESCs. The XO ESCs (*Rbmy1a1*, *#2*; *Ssty2*, *#3*) showed one copy of X with Y absent. *WT,* untreated mouse. *Vertical axis*, copy number; *horizontal axis*, chromosome number

We initially examined whether complete elimination of a chromosome could be achieved efficiently by using CRISPR/Cas9-mediated multiple cuts at chromosome-specific sites. First, we examined whether the mouse Y chromosome contains unique repeated sequences that could be used for large-scale chromosomal editing via short-guide RNAs (sgRNAs), and whether such editing could result in Y chromosome deletion. Sequence analysis for all mouse chromosomes, using 23-bp sgRNA target sequences containing an adjacent ‘NGG’ protospacer adjacent motif (PAM), showed that each chromosome indeed has unique and multiple repeated sequences for targeting by a single specific sgRNA (Additional file 1: Table S1 and Additional file 2: Table S2). These repeated sequences appeared either clustered at one region or scattered across the entire chromosome (Fig. 1a).

To examine whether chromosome deletion could be achieved directly by CRISPR/Cas9 editing in established mouse embryonic stem (ES) cells, we designed a sgRNA that targeted the locus consisting of more than 50 repeats of an RNA-binding motif gene on the Y chromosome (*Rbmy1a1*), which are clustered in the short arm [10]. Alternatively, we targeted the spermiogenesis-specific transcript on Y 2 (*Ssty2*) [11] that contains repeated gene sequences scattered in the long arm (Fig. 1a). One day after transfecting mouse ES cells of XY genotype with plasmids expressing Cas9, Y chromosome-targeting sgRNAs, and mCherry, we sorted mCherry-positive ES cells by FACS and cultured them on feeder cells (Fig. 1b). To detect whether the Y chromosome was eliminated, we performed DNA-FISH (fluorescence in situ hybridization) analysis 3 days later using a whole-chromosome probe for the Y chromosome and near-centromere probe XqA7.3 for the X chromosome (see “Methods”) on the transfected cells. We found that about 30 and 50% of ES cells targeted for *Rbmy1a1* and *Ssty2*, respectively, had no Y chromosome signals, indicating Y chromosome elimination in some culture cells (Fig. 1c, d). This efficiency of chromosome deletion was much higher than that achieved via spontaneous chromosome loss or Cre-mediated chromosome deletion in previous studies (<10^−4^) [2, 3]. To further confirm Y chromosome elimination, single clones derived from transfected cells were randomly picked, expanded, and genotyped. We found the absence of the Y-specific gene *Sry* (on the short arm of the Y chromosome) in 4/18 (22%) clones with *Ssty2* targeting and 10/52 (19%) clones with *Rbmy1a1* targeting (Fig. 1e, f). Karyotyping of *Sry*-negative ES cells showed 39 instead of 40 chromosomes (Fig. 1g; Additional file 1: Table S3), and DNA FISH and whole genome sequencing (WGS) further confirmed the complete deletion of the Y chromosome (Fig. 1c, h, i).

To test whether the Y chromosome could be eliminated in vivo by CRISPR-Cas9 editing, we delivered the sgRNA-*Ssty2*-EGFP construct targeting the Y chromosome to E14.5 mouse brain via in utero electroporation (Additional file 1: Figure S1a). Two days after electroporation, we sorted EGFP-positive cells in the male brain by FACS and performed DNA-FISH (Additional file 1: Figure S1b). We found that about 40% of EGFP-positive cells showed no Y chromosome signal (Additional file 1: Figure S1c–e). By contrast, only 1% of wild-type (WT) cells and 8% of EGFP-negative cells in the brain contained no Y chromosome signal (Additional file 1: Figure S1c–e). These results indicate that the Y chromosome could be efficiently eliminated in vivo.

Together, these results indicate that the Y chromosome could be selectively eliminated in vitro and in vivo by CRISPR/Cas9-mediated multiple cuts at chromosome-specific repeated sequences.

### Generation of a mouse model with Turner syndrome by Y chromosome elimination

**Fig. 2:**
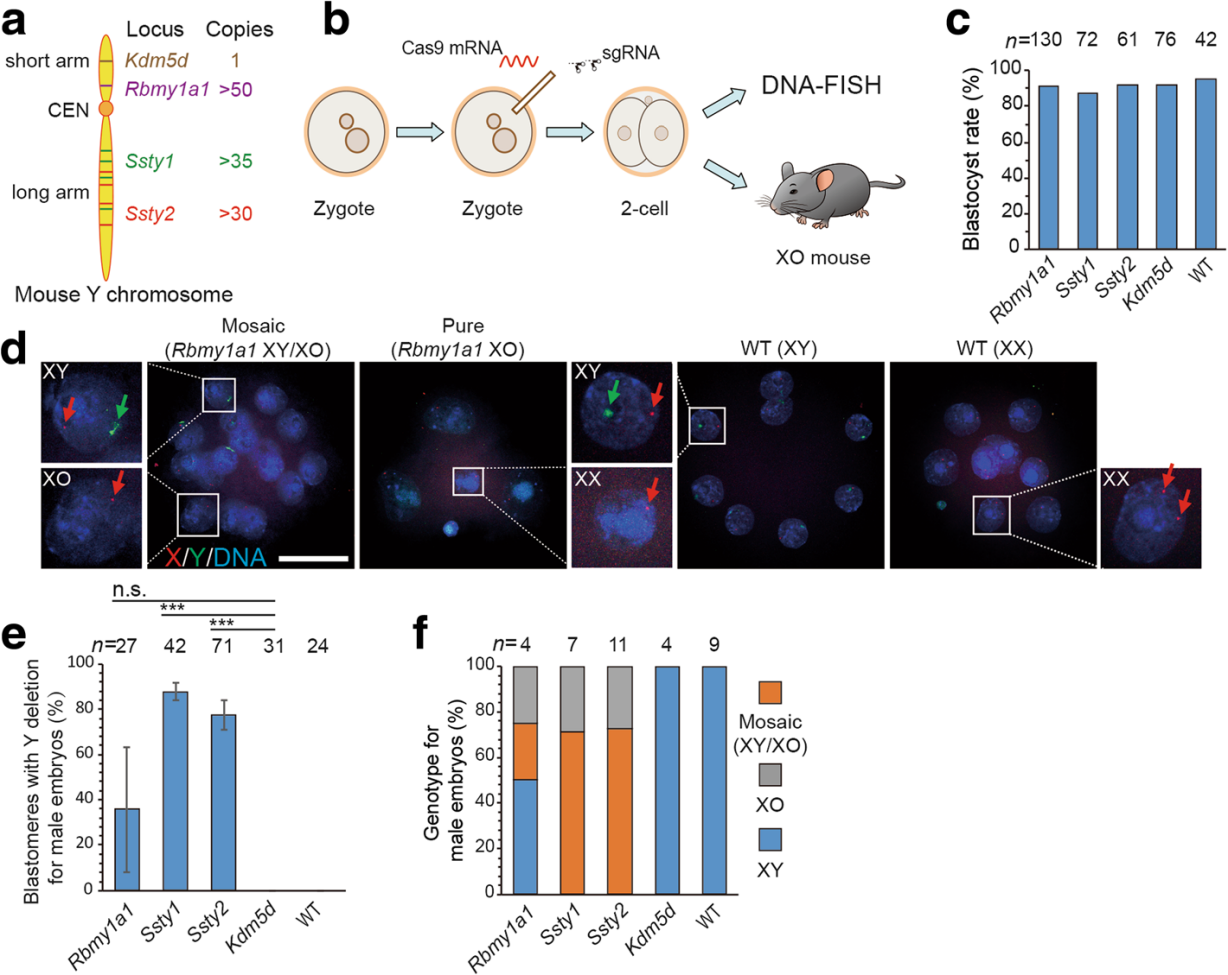
Elimination of the Y chromosome in zygotes by CRISPR/Cas9-mediated gene editing. **a** Targeted gene loci in the Y chromosome: *Rbmy1a1*, clustered in the short arm; *Ssty1*, *Ssty2*, scattered in the long arm; *Kdm5d*, control gene. **b** Experimental design. Cas9 mRNA and two specific sgRNAs that targeted the *Rmby1a1*, *Ssty1*, *Ssty2*, or *Kdm5d* locus were injected into individual mouse zygotes, which were further cultured to 4- to 16-cell embryos for DNA-FISH analysis or transferred into recipients. WT, embryos without injection of Cas9 mixture. **c** Blastocyst rate of embryos generated by gene editing. The experimental group (*Rbmy1a1*, *Ssty1*, *Ssty2*) showed no difference compared to the control group (*Kdm5d* or WT); *n* is the sample size of injected embryos. **d** Representative DNA-FISH analysis of 4- to 16-cell embryos after *Rbmy1a1* targeting. *Green*, FITC-labeled whole-chromosome probe for Y chromosome; *red*, Texas red-labeled X chromosome probe for XqA7.3; *blue*, Hoechst 33342-labeled DNA. *Green arrows*, Y; *red arrows*, X. WT male embryo (XY), one green signal and one red dot; WT female embryo (XX), two red dots, no green signal; pure XO embryo (XO), one red dot, no green signal; mosaic embryo (XY/XO), co-existing XO and XY genotype in the blastomeres from the same embryo. *Insets*: single blastomeres shown at higher resolution. *Bar*, 50 μm. **e**, **f** Results of DNA-FISH analysis of the ratio of blastomeres with Y deletion (**e**) and sex chromosomal genotype (**f**) in each male embryo at 4- to 16-cell stage. Experimental group, *Rbmy1a1*, *Ssty1*, *Ssty2*; control group, *Kdm5d* and WT (***P < 0.001, not significant > 0.05, *t* test). *n* represents the sample size of blastomeres in **e** and sample size of embryos in **f**. *Error bars* represent SEM

Next we examined whether this method could be applied to generate animal models for aneuploidy, such as Turner syndrome [1]. We first injected Cas9 mRNA and two specific sgRNAs that targeted the *Rmby1a1*, *Ssty1*, or *Ssty2* locus into individual mouse zygotes, and injected zygotes were then cultured to the blastocyst stage (Fig. 2a, b). Gene-edited embryos showed normal development compared to untreated embryos (without injection of Cas9 mixture) or embryos treated with two sgRNAs targeting only a single-copy gene (*Kdm5d*) on the Y chromosome, with a similar blastocyst rate (Fig. 2c). To detect whether the Y chromosome was indeed eliminated, we performed DNA-FISH analysis on injected embryos at the 4- to 16-cell stage. We focused only on male embryos, which were determined by the presence of only a single fluorescent dot for the X chromosome in each blastomere. A green fluorescent signal for the Y chromosome probe was absent in some blastomeres of injected male embryos, suggesting the Y chromosome had been eliminated (Fig. 2d). The efficiency of Y chromosome elimination varied from 40 to 90% among the blastomeres of male embryos from three sets of experiments targeted at three different targeted gene loci (Fig. 2e). Based on the extent of Y chromosome deletion in all blastomeres, the injected male embryos could be classified into three phenotypes: XY (no Y deletion), pure XO (Y deletion in all blastomeres), and XY/XO (Y deletion in some blastomeres) (Fig. 2f). These results indicate that complete or mosaic Y chromosome elimination in mouse embryos could be achieved by this method.

**Fig. 3:**
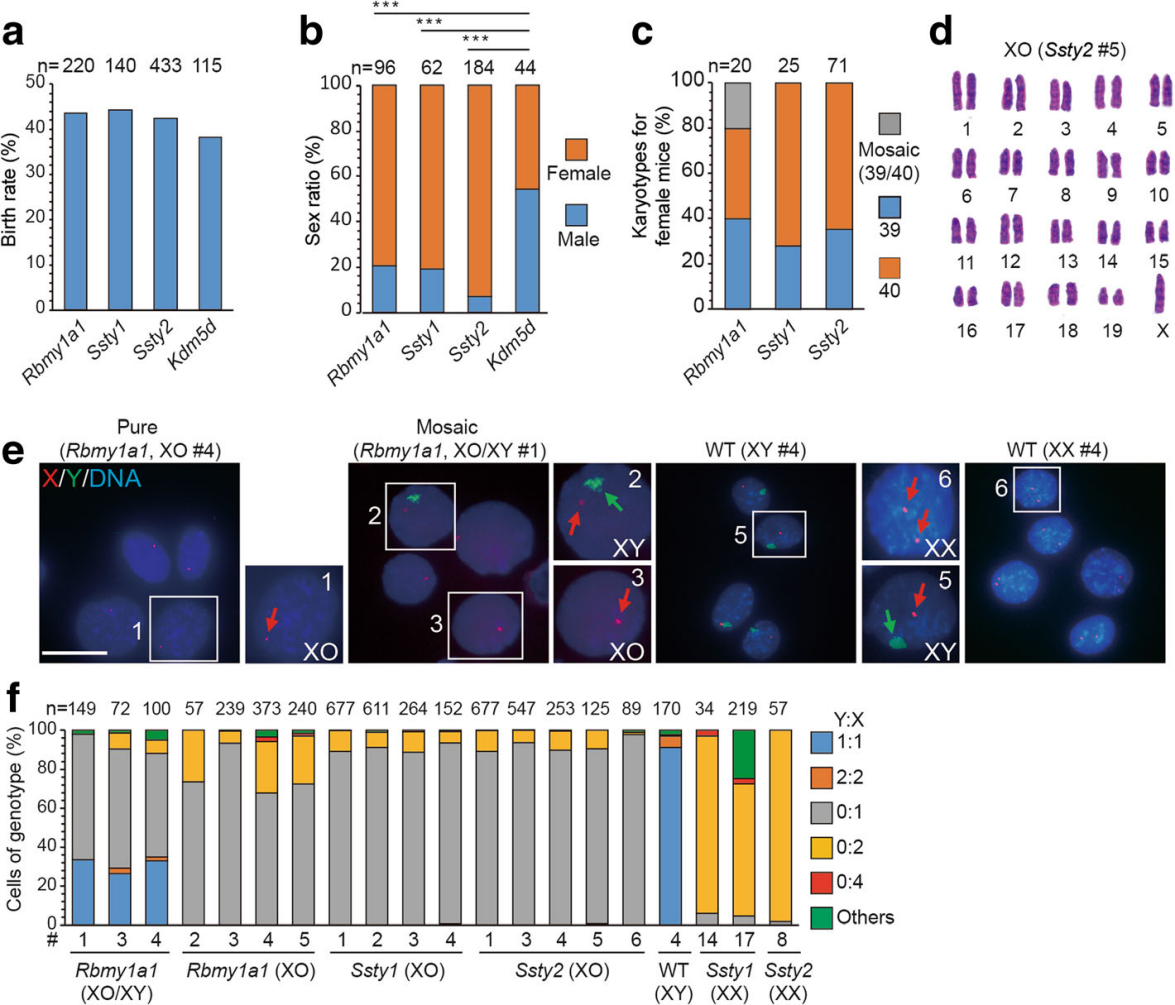
Generation of mouse model with Turner syndrome by Y chromosome elimination. **a** Birth rate of gene-edited embryos for Y chromosome elimination. The experimental group (*Rbmy1a1*, *Ssty1*, *Ssty2*) showed no significant difference compared to the control group (*Kdm5d*). *n* is the sample size of transferred embryos. **b** Sex ratio of mice generated by gene editing. The experimental group (*Rbmy1a1*, *Ssty1*, *Ssty2*) showed more mice with female gonads than the control group (*Kdm5d* or dCas9 and sgRNA targeting *Ssty2*). *n* is the sample size of mice generated by gene editing (***P < 0.001, **P < 0.01, *P < 0.05, Chi-square test). **c** Percentage of female mice with different karyotypes. *n* is the sample size of female mice. **d** Representative image of the XO karyotype of a female generated by *Ssty2* targeting. **e** Representative DNA-FISH images of cultured tail fibroblasts derived from female mice with *Rbmy1a1* targeting, showing XO and XO/XY genotypes. Untreated (WT), male or female mice without gene editing. *Green*, Y probe; *red*, X probe for XqC3; *blue*, DNA. *Green arrows*, Y; *red arrows*, X. *Numbered squares*, single cells shown at a higher resolution in the *right panel. Bar*, 20 μm. **f** Results of DNA-FISH analysis on the gene-edited female mice. Percentages of cells (including dividing cells, Y:X = 2:2 or 0:2) exhibiting different genotype ratios. Data include XO mice (*Rbmy1a1*, mice 2 to 5; *Ssty1*, mice 1 to 4; *Ssty2*, mice 1 to 5; see Additional file 1: Table S3 for corresponding mice) as well as mosaic XO/XY mice (*Rbmy1a1*, mice 1, 3, and 4). *n* is the sample size of counted cells

**Fig. 4:**
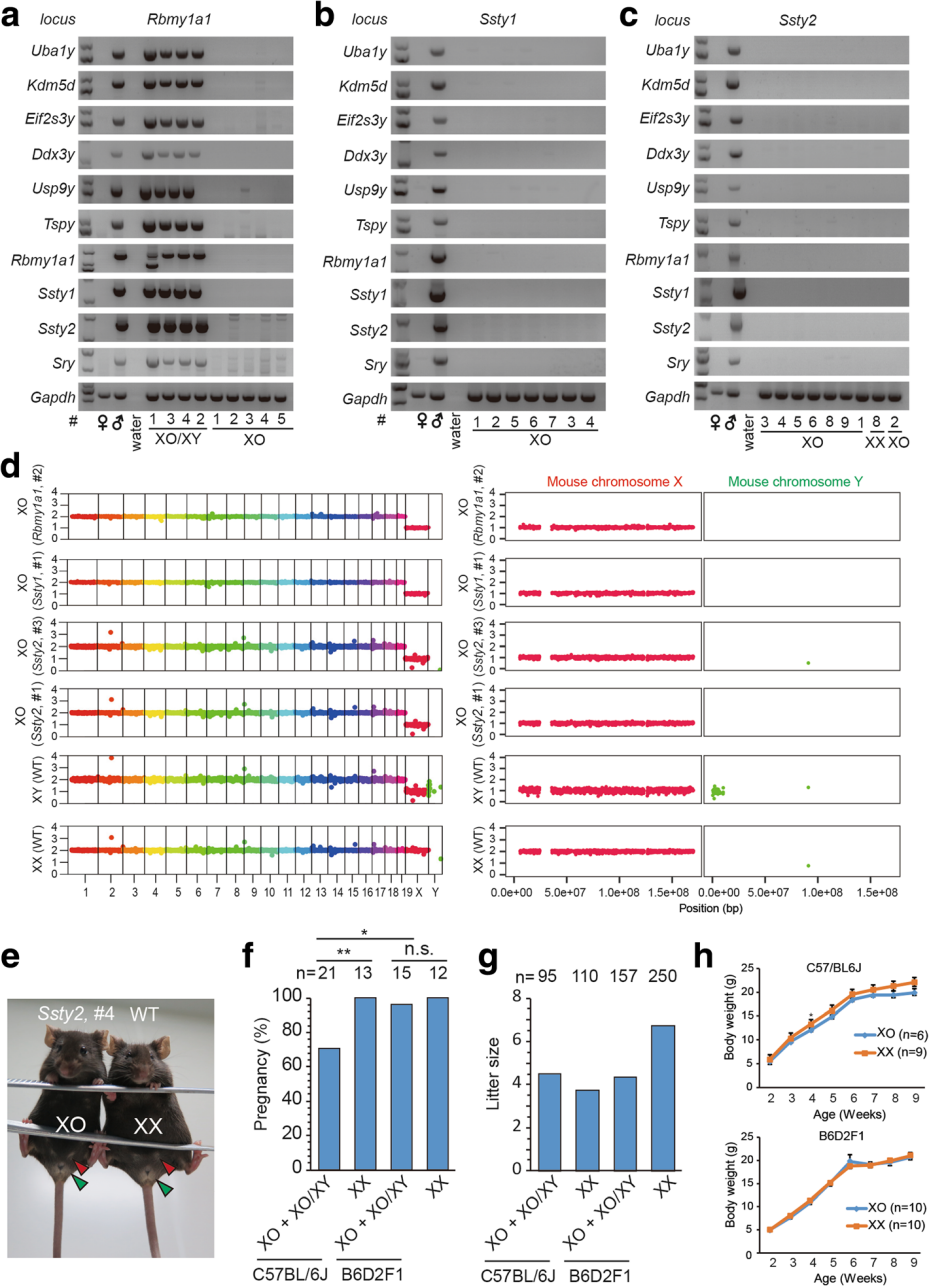
Identification of a mouse model with Turner syndrome generated by Y chromosome elimination. **a**–**c** *Rbmy1a1* (**a**), *Ssty1* (**b**), *Ssty2* (**c**) targeted mice used for genotyping (listed in Additional file 1: Table S3). The XO pure mice showed no Y chromosome-specific genes, suggesting complete elimination of the Y chromosome. Y chromosome short arm, *Uba1y*, *Kdm5d*, *Eifs23y*, *Dxd3y*, *Usp9y*, *Tspy*, *Sry*, *Rbmy1a1*; Y chromosome long arm, *Ssty1*, *Ssty2. Gapdh*, control gene in autosome. **d** WGS showed Y chromosome elimination of XO mice. Histograms of X and Y chromosome are shown in the *right panel* at a higher resolution. The XO mice (*Rbmy1a1, #2* and *Ssty1, #1*) showed one copy of the X chromosome with the Y chromosome absent. *Vertical axis*, copy number; *horizontal axis*, chromosome number. **e** Twelve-week old XO mouse from *Ssty2* XO #4 showing normal female genitals (*green arrowheads*) and nipples (*red arrowheads*). WT, female mice without gene editing. **f**, **g** Fertility of mice with Y chromosome deletion. The gene-edited XO female mice (number indicated above) were paired with wild-type male mice for over 3 months. The frequency of pregnancy (**f**) and litter size (**g**) was determined and compared with those of XX siblings (**P < 0.01, *P < 0.05, not significant (*n.s.*), P > 0.05, Chi-square test). **h** The weight of XO mice (*Rbmy1a1*, *Ssty1*, *Ssty2*) and the siblings of XX mice. The mice were measured about once a week from 1 week to 9 weeks. Means ± SEM

In parallel to the above studies, we transferred the injected zygotes into recipient female mice and obtained newborn mice at similar birth rates compared to control embryos, indicating no developmental defect was induced by the gene editing (Fig. 3a). Interestingly, most of the newborn mice were female, as judged by the presence of female genitals and nipples, with the percentage of females ranging from 79–90% in experiments targeting three different gene loci (Fig. 3b). As a control, mice generated by Cas9 and sgRNA targeting *Kdm5d* or dCas9 (nuclease-dead Cas9) and sgRNA targeting *Ssty2* exhibited normal sex ratios (Fig. 3b). To test whether some of these female mice with *Rbmy1a1*, *Ssty1*, or *Ssty2* targeting were derived from male embryos that were transformed into females via Y chromosome elimination, we performed karyotyping of tail tissues or bone marrow of all female mice generated by gene editing and found that 26–60% of these mice indeed had 39 instead of 40 chromosomes (Fig. 3c, d; Additional file 1: Table S3). DNA-FISH analysis further confirmed that the missing chromosome was indeed the Y chromosome (Fig. 3e). Furthermore, FISH analysis also showed that some mice exhibited XO and XY phenotypes in different cells of the same mouse, indicating mosaicism in Y deletion (Fig. 3e, f). Further confirmation of complete Y chromosome elimination in XO mice was provided by PCR genotyping, which showed the absence of ten chromosome-specific genes, located in both the short- and long-arm (Fig. 4a, c). In addition, WGS of XO mice also confirmed complete elimination of the Y chromosome (Fig. 4d). Together, these results showed that the Y chromosome could be efficiently eliminated by CRISPR/Cas9-mediated targeting on clustered repeated gene sequences of *Rmby1a1* or scattered repeated gene sequences of *Ssty1* or *Ssty2*.

Compared to the siblings with the XX karyotype, XO mice obtained by our gene-editing approach showed normal body weight (Fig. 4e, h; Additional file 1: Table S4), consistent with previous reports [12–14]. However, XO mice or XO/XY mosaic mice from an inbred C57BL/6 background showed reproductive defects compared to their wild-type counterparts, including the frequency of pregnancy and parturition (Fig. 4f, g; Additional file 1: Table S4), all of which are found in patients with Turner syndrome [15, 16]. Interestingly, many patients with aneuploidy diseases (e.g., Turner syndrome) often show mosaicism [17]. Our approach is an efficient way to generate aneuploidy mouse models with mosaicism, which is not found in previous models [18].

**Fig. 5:**
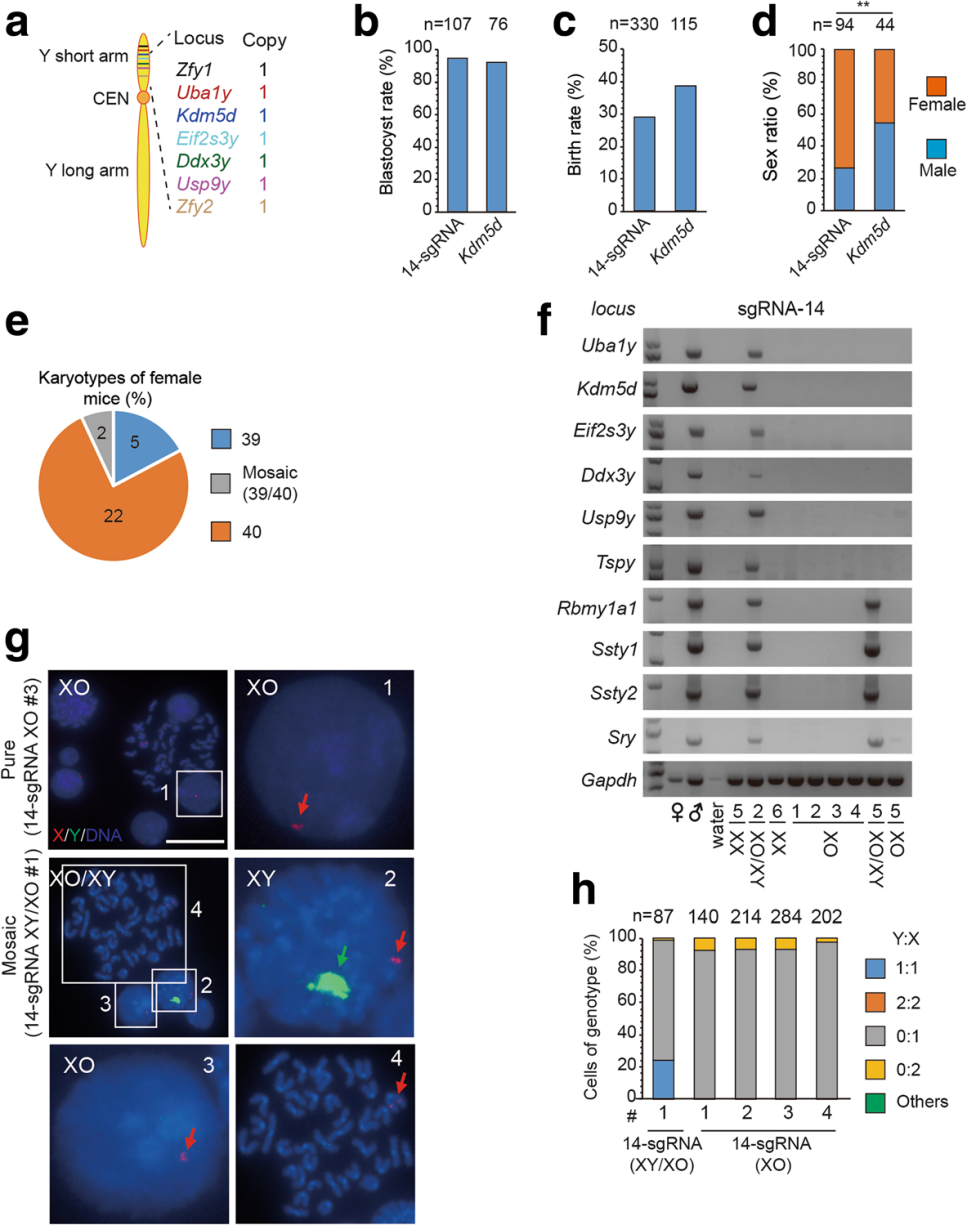
Elimination of mouse Y chromosome in zygotes with an sgRNA cocktail. **a** Schema of targeted gene loci in Y chromosome. 7 one-copy genes, each targeted with two sgRNAs, are in the short arm. **b**, **c** The blastocyst (b) and birth rate (c) of embryos with 14-sgRNA cocktail for Y chromosome elimination. Control: *Kdm5d.* ‘n’: sample size. **d** Sex ratio of the mice generated by gene editing. Experimental group (14-sgRNA cocktail) showed more female mice than control group (*Kdm5d*). (***P < 0.001, **P < 0.01, *P < 0.05, Chi-square test). **e** Karyological characteristics of the female mice. **f** Genotyping analysis of the female mice obtained by 14-sgRNA targeting. The mice used for genotyping were listed in Additional file 1: Table S3. The mosaic mouse of 14-sgRNA #2 showed the integrity for the remaining Y chromosome. In contrast, the mosaic mouse of 14-sgRNA #5 showed the large deletion of short arm in the remaining Y chromosome. The XO pure mice showed no Y chromosome-specific genes, suggesting complete elimination of whole Y chromosome. **g** Representative DNA-FISH analysis of the female from 14-sgRNA cocktail targeting. The pure XO mouse of 14-sgRNA-XO #3 showed the absence of Y chromosome. The mosaic mouse of 14-sgRNA-XY/XO #1 showed co-existing XO and XY genotype in the cells from the same mouse. Green: a whole Y chromosome probe; red: X; blue: DNA. Arrows: Y or X chromosome. Numbered squares: single bone marrow cells shown at a higher resolution on the right or down panel. Bar, 20 μm. **h** Stacked bar graphs of data from DNA-FISH analysis of gene-edited female mice. Percentages of cells (including dividing cells, Y:X = 2:2 or 0:2) exhibiting different ratio of genotype. Experimental group: 14-sgRNA-XO #1 to #3; 14-sgRNA-XY/XO #1, #2. Control male mice: (WT-XY #4); control female mice: (*Ssty1*-XX #14, *#*17, *Ssty2*-XX #8). ‘n’: sample size of counted cells

The above results on Y chromosome deletion were obtained by using two sgRNAs that target repeat sequences. To generalize this method, we further explored whether the Y chromosome could also be selectively eliminated in zygotes with multiple sgRNAs, each targeting a chromosome-specific single-copy sequence. For this purpose, we designed 14 sgRNAs targeting the short-arm of the Y chromosome (Fig. 5a) and injected Cas9 mRNA together with a cocktail of these 14 sgRNAs into mouse zygotes. We found that 94% of embryos reached the blastocyst stage and 29% of embryos yielded live births after they were transferred into pseudo-pregnant mice, with 73% (69/94) female (76% showed XX karyotypes, 17% showed pure XO karyotypes, and 7% showed XY/XO karyotypes) (Fig. 5b–e; Additional file 1: Table S3), as confirmed by genotyping and DNA-FISH (Fig. 5f–h; Additional file 1: Table S3). Thus, the Y chromosome could also be selectively eliminated in zygotes by CRISPR/Cas9-mediated multiple-sgRNA targeting at chromosome-specific single-copy sequence sites. This approach offers a potential way to use chromosome-specific single nucleotide polymorphisms for chromosome removal without affecting homologous chromosome [19].

### Generation of mouse model with Turner Syndrome by X chromosome elimination

**Fig. 6:**
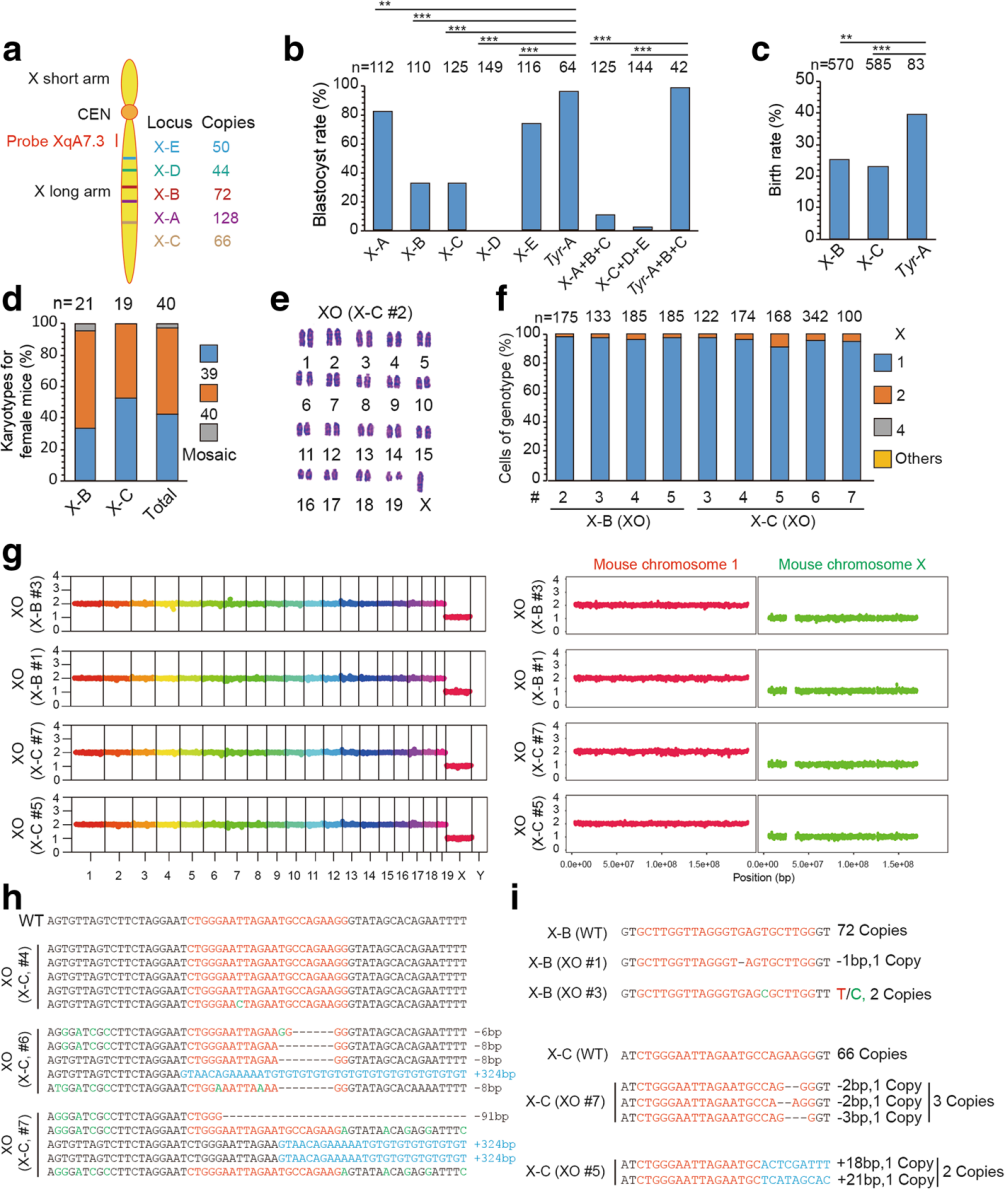
Generation of mouse model with Turner syndrome by X chromosome elimination. **a** Targeted loci in the X chromosome. Five sgRNA target sequences (from X-A to X-E) are X chromosome-specific repeated sequences in non-coding regions. Texas red-labeled probe XqA7.3 is near the centromere. **b** Blastocyst rate of embryos generated by gene editing for X chromosome elimination. Embryos edited using triple sgRNAs (X-A + B + C or X-C + D + E) showed embryonic lethality, whereas those edited using a single sgRNA (X-A, X-B, X-C, or X-E) reached the blastocyst stage, with a lower blastocyst rate than the control group (*Tyr*-A or *Tyr*-A + B + C). *Tyr*-A or *Tyr*-A + B + C, a single sgRNA or triple sgRNAs targeting the locus of *Tyr*, a coat color gene with a single copy on the autosome. *n* is the sample size of cultured embryos (***P < 0.001, **P < 0.01, *P < 0.05, Chi-square test). **c** Birth rate of embryos gene-edited for X chromosome elimination. The experimental group (X-B, X-C) showed a lower birth rate than the control group (*Tyr*-A). *n* is the sample size of transferred embryos (***P < 0.001, **P < 0.01, *P < 0.05, Chi-square test). **d** Percentage of female mice (X-B, X-C) with different karyotypes. Percentages of cells (including dividing cells, X = 4) exhibiting different genotype ratios. *n* is the sample size of female mice. **e** Representative image for XO karyotype of a female generated by X-C targeting. **f** Representative DNA-FISH analysis of the XO mice from X-B and X-C targeting. **g** WGS showing X chromosome elimination of XO mice. The histograms of chromosome 1 and X are shown in the *right panel* at a higher resolution. The XO mice (X-B, mice 1 and 3; X-C, mice 5 and 7) showed one copy of the X chromosome. *Vertical axis*, copy number; *horizontal axis*, chromosome number. **h** The targeted loci of XO mice (X-C, mice 4, 6, and 7) were PCR amplified and pMD-19 T TA cloned for sequencing. Indels occurred in two of three XO mice. **i** WGS mapping revealed that most copies of clustered repeat sequences by X-B or X-C targeting were deleted in XO mice. For X-B targeting, 72 copies of target sequences were detected in WT mice. By contrast, only one copy and two copies of target sequence were detected with mutations in X-B XO #1 and X-B XO #3, respectively. For X-C targeting, 66 copies of target sequences were detected in WT mice (untreated mice), but only two and three copies of target sequences were detected with mutations in X-C XO #5 and X-C XO #7, respectively

Next, we examined whether the same CRISPR/Cas9-mediated genome editing could be used to eliminate the X chromosome. We injected Cas9 mRNA together with a single sgRNA (X-A to X-E) or triple sgRNAs (X-A + B + C and X-C + D + E) that targeted at X chromosome-specific repeated sequences in non-coding DNA sequences (Fig. 6a). We found that triple sgRNAs editing led to serious embryonic lethality, possibly due to large fragment deletion of X chromosomes, whereas single sgRNA targeting yielded some embryos that reached the blastocyst stage (Fig. 6b). We then transferred 2-cell embryos edited with the single sgRNA (X-B, X-C) to recipient female mice and found that these edited embryos yielded birth rates lower than control embryos, using sgRNA that targets at *Tyr*, a coat color gene with a single copy on autosome (Fig. 6c), suggesting that this gene editing may have induced developmental defects, possibly involving elimination of the single X chromosome in some male embryos or both X chromosomes in some female embryos. Gene-edited newborn female mice consisted of 42.5% XO mice, 55% XX mice and 2.5% XO/XX mice (Fig. 6d, e and Additional file 1: Table S3). The absence of X chromosome in some female mice was confirmed by DNA-FISH and WGS (Fig. 6f, g). As expected, indels or large deletions indeed occurred at targeted non-coding sequences in the remaining X chromosome in XO mice (Fig. 6h, i), but these deletions may not induce obvious deleterious effect (Additional file 1: Figure S2a). In principle, these indels and large deletions could be avoided by sgRNAs that target at only one of the two homologus chromosomes, based on single nucleotide polymorphism. Thus, selective X chromosome elimination could also be achieved by CRISPR/Cas9-mediated gene editing at chromosome-specific repeated sequences, suggesting that this approach could be used for elimination of chromosomes in general.

In addition to establishing a chromosome-deleted mouse model, we have also derived ES cells from blastocysts that were gene-edited by single sgRNA for X chromosome elimination. We obtained 25 out of 52 ES cell lines without a Y chromosome, and examined 10 out of 25 female ES cell lines for further karyotyping. We found that two of them showed a pure XO karyotype, as confirmed by DNA-FISH (Additional file 1: Figure S2b, d). These ES cell lines with distinct karyotypes could be useful for studying the effect of chromosome deletion at the cellular level.

### CRISPR/Cas9-mediated gene editing promotes autosome elimination

**Fig. 7:**
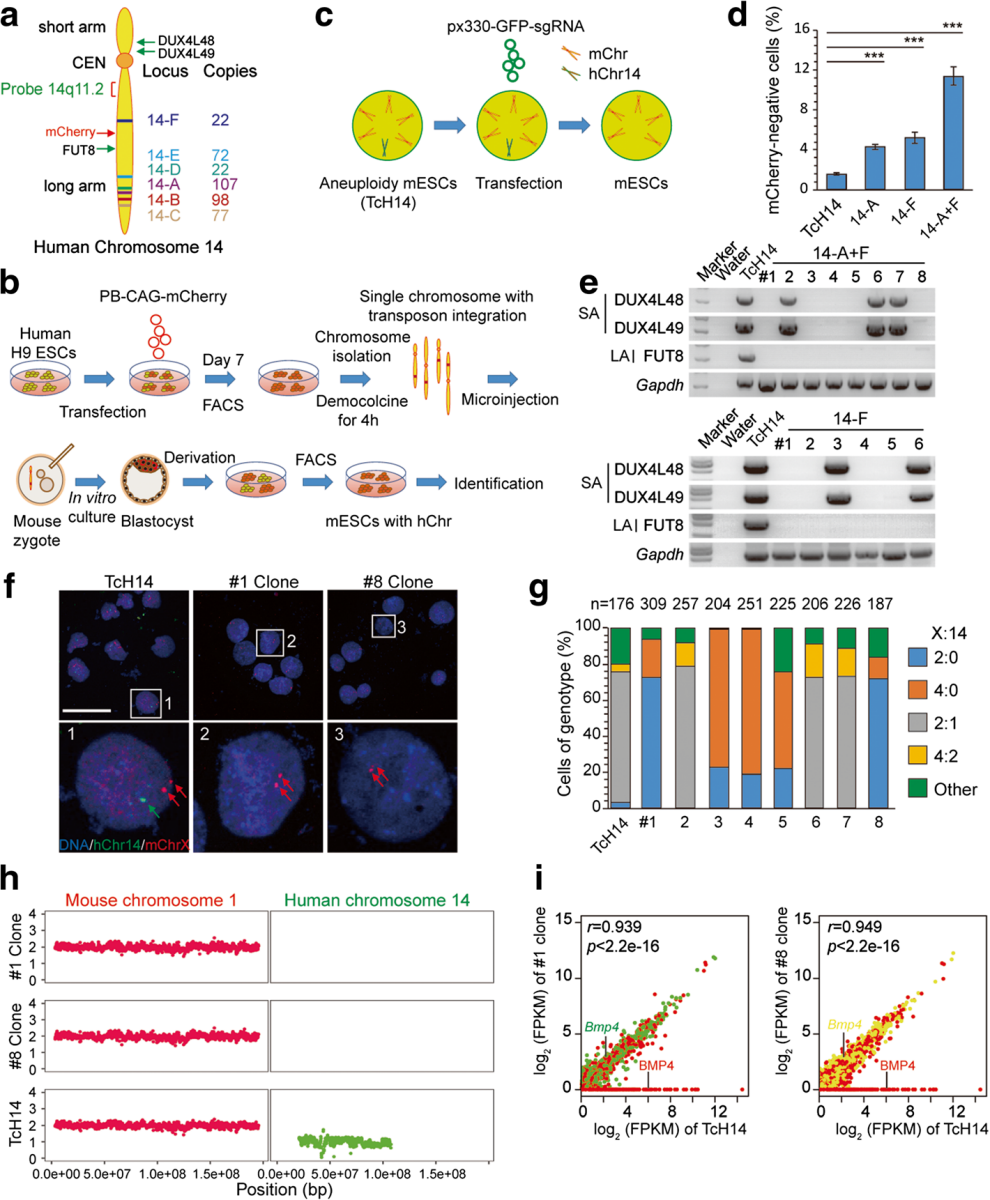
Elimination of human chromosome 14 in cells by CRISPR/Cas9-mediated gene editing. **a** Targeted loci in human chromosome 14. Six sgRNA target sequences (from 14-A to 14-F) are specific for human chromosome 14 repeated sequences in the non-coding regions. FITC-labeled probe 14q11.2 is near the centromere. *Red arrow*, insertion site of PB-CAG-mCherry; *green arrows*, gene loci for genotyping. **b** Establishment of aneuploid mouse ES cells with hChr14 (TcH14). **c** Experimental design. Aneuploid cells were transfected with plasmids expressing Cas9, chromosome targeting sgRNAs, and mCherry. One day later, GFP-positive ES cells were sorted by FACS and cultured in six wells for DNA-FISH analysis or 96 wells for single cell cloning and genotyping. **d** Percentage of mCherry-negative cells after gene editing. TcH14 cells were transfected with px330 plasmid containing different sgRNAs and then sorted by FACS 1 day later. Three days later, transfected TcH14 cells were analyzed by FACS. Data are presented as means ± SEM (n = 3, ***P < 0.001, **P < 0.01, *P < 0.05, Chi-square test). **e** Genotyping analysis of mCherry-negative cell lines derived from gene editing on TcH14 cells. Note that hChr14 in the cell lines (14-A + F #2, #6, #7; 14-F #3, #6) were partial deletions. **f** Representative DNA-FISH analysis of clones #1 and #8 from 14-A + F targeting. *Green*, human chromosome 14 probe for 14q11.2; *red*, X probe for XqA7.3; *blue*, DNA; *green arrow*, human chromosome 14; *red arrows*, mouse X chromosome. *Numbered squares*, single cells shown at higher resolution in the *bottom panels. Bar*, 50 μm. **g** Results of DNA-FISH analysis on mCherry-negative clones from 14-A + F targeting. Percentages of cells (including dividing cells, X:14 = 4:2 or 4:0) exhibiting different X:14 ratios. Data include TcH14 mCherry-negative clones (14-A + F #1 to #8) as well as control TcH14 mCherry-positive clones. *n* is the sample size of counted cells. **h** The weight of XO mice (X-B, n = 3; X-C, n = 3) and the siblings of XX mice (n = 6) was measured about once a week from 1 to 9 weeks. Means ± SEM. **i** RNA-seq analysis of TcH14 cells and cells with chromosome correction. The corrected cell lines (14-A + F #1 and #8) showed no gene expression on hChr14 (BMP4 for example)

**Fig. 8:**
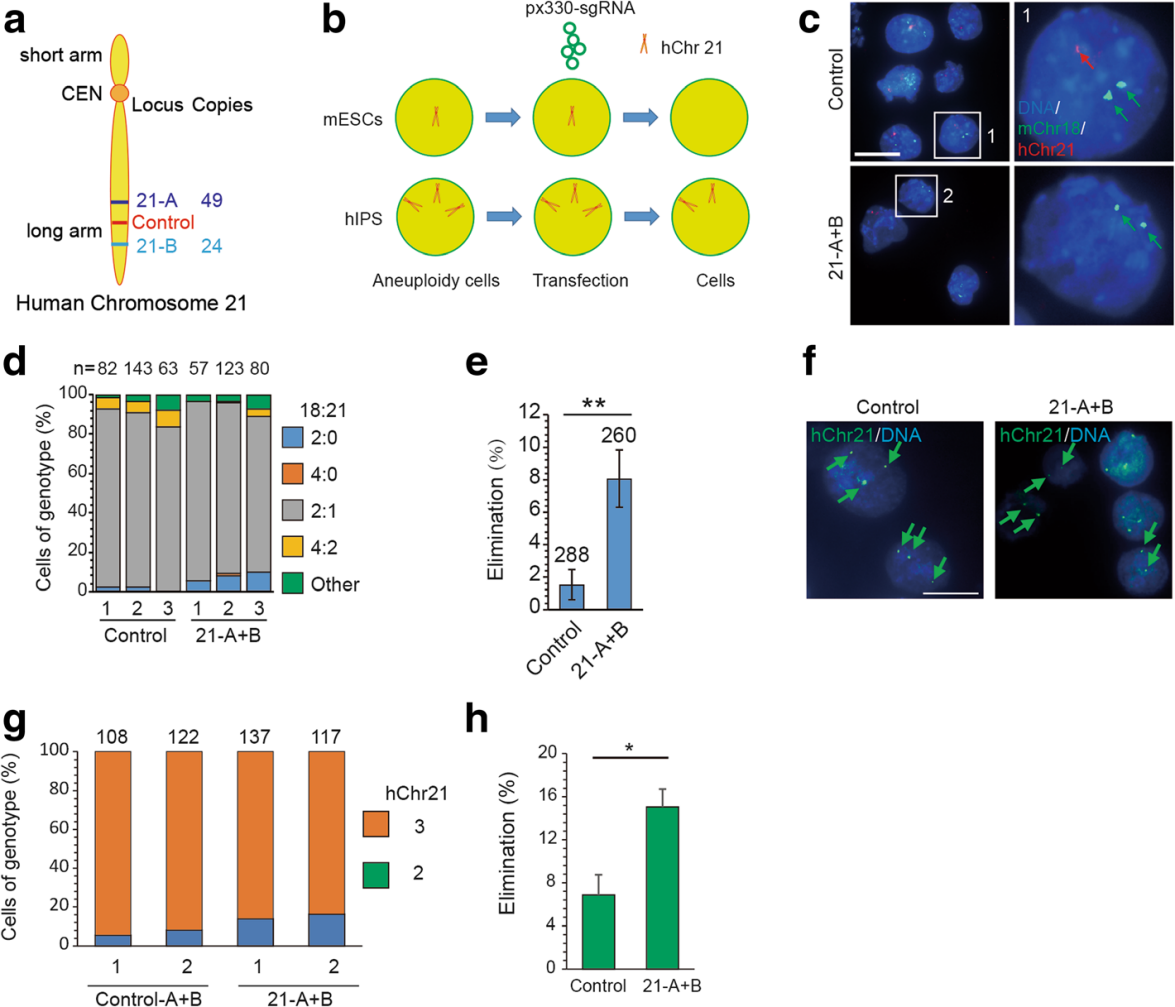
Elimination of human chromosome 21 in mouse aneuploid cells and DS iPS cells by CRISPR/Cas9-mediated gene editing. **a** Targeted loci in hChr21. Two sgRNA target sequences (21-A and 21-B) are specific for hChr21 repeated sequences in the non-coding regions. Control sgRNAs (21-NC-A and 21-NC-B) are hChr21-specific sequences with a unique target site. **b** Experimental design. Aneuploid cells were transfected with plasmids expressing Cas9, chromosome-targeting sgRNAs, and GFP. One day later, GFP-positive ES cells were sorted by FACS and cultured in six wells for DNA-FISH analysis. **c** Representative DNA-FISH analysis of mixed mouse ES cells targeted at either 21-A + B or control sgRNAs. *Red*, Texas red-labeled whole-chromosome probe for chromosome 21; *green*, FITC-labeled mouse chromosome 18qA1 probe; *blue*, Hoechst 33342-labeled DNA. *Green arrows*, mChr18; *red arrow*, hChr21. *Numbered squares*, single cells shown at higher resolution in the *right panels. Bar*, 20 μm. **d** Results of DNA-FISH analysis on TcH21 cells targeted at either 21-A + B or control sgRNAs (two sgRNAs with one targeting site on hChr21). Percentages of cells (including dividing cells) exhibiting different mChr18: hChr21 ratios. *n* is the sample size of counted cells. **e** Efficiency of hChr21 elimination based on DNA-FISH analysis after TcH21 cells targeted at either 21-A + B or control sgRNAs (n = 3, **P < 0.01, *t* test). **f** Representative DNA-FISH analysis of human iPSCs with trisomy 21 targeted at either 21-A + B or control sgRNAs. *Green*, FITC-labeled hCHr21 CEN probe; *blue*, Hoechst 33342-labeled DNA. *Arrows*, hChr21. *Bar*, 20 μm. **g** Results of DNA-FISH analysis on TcH21 cells targeted at either 21-A + B or control sgRNAs (two sgRNAs with one targeting site on hChr21). Percentages of cells (including dividing cells) exhibiting different mChr18: hChr21 ratios. *n* is the sample size of counted cells. **h** Efficiency of hChr21 elimination based on DNA-FISH analysis after DS iPSCs targeted at either 21-A + B or control sgRNAs (n = 2, **P < 0.01, Chi-square test)

We next tested whether an extra chromosome in aneuploid cells could be eliminated by CRISPR/Cas9 editing. We focused on an ES cell line with an extra human chromosome 14 (hChr14), which was established by chromosome transfer (Fig. 7a, b; see “Methods”) and known to be stable in cell lines [20]. After FACS analysis, we found that 1.6% of the mouse ES cells with hChr14 (termed TcH14) exhibited hChr14 loss during every passage (Fig. 7c, d). Using sgRNAs (14-A to 14-F) targeted at repeated sequence sites, we were able to achieve complete elimination of hChr14 in up to 15% of cells, as indicated by the absence of mCherry expression (Fig. 7c, d). Next, we performed PCR genotyping and DNA-FISH analysis on mCherry-negative clones. We found five out of eight clones from 14-A + F targeting and four out of six clones from 14-F targeting showed complete deletion of hChr14 (Fig. 7e–g). By contrast, clones 14-A + F #2, #6, and #7 and clones 14-F #3 and #6 showed the existence of genes in the short arm and hChr14 DNA-FISH probe, indicating incomplete deletion of hChr14 (Fig. 7f, g). Further genotype evidence of the hChr14 deletion was confirmed by WGS, as well as the expression profile of genes unique to hChr14 (Fig. 7h, i). RNA-seq analysis revealed that all hChr14-specific genes showed no expression in clones 14-A + F #1 and #8. By contrast, ES cells with hChr14 showed a normal expression profile of genes unique to hChr14 (Fig. 7i). In addition, by injecting these aneuploid cells into oocytes and then injecting Cas9 mRNA and sgRNAs 6 h later, we found that 13% of gene-edited blastocysts showed no mCherry signal, indicating complete deletion of hChr14 (Additional file 1: Figure S3a–c). Moreover, we found this method could also be applied to promote human chromosome 7 (hChr7) loss in human cancer cell line HT-29, which contains four hChr7s in most cells (Additional file 1: Figure S4), and extra human chromosome 21 (hChr21) loss in aneuploid mouse ES cell lines (Fig. 8a–e) derived from mice with Down syndrome (DS; Tc1) [21]. Notably, we observed CRISRP/Cas9-mediated multiple DNA cleavages could also produce chromosome rearrangement in cancer cells (Additional file 1: Figure S4e). Finally, we tested whether hChr21 could be selectively deleted via this approach in human iPSCs with trisomy 21 derived from DS patients (ATCC® ACS-1003™). DS iPS cells were transfected with two sgRNAs (21-A and 21-B, containing 49 and 24 cleavage sites, respectively) targeting hChr21-specific repeated sequence sites (Fig. 8a, b). Transfected cells were sorted and analyzed by DNA-FISH with a centromere (CEN) probe for hChr21. We found that 15.0% of cells showed two hChr21 probe signal dots (Fig. 8f–h). As a control, only 6.9% of cells transfected with sgRNA containing only one cleavage site on hChr21 showed two hChr21 probe signal dots (Fig. 8f–h).

Taken together, these results indicate that multiple CRISPR/Cas9-induced DNA cleavages could promote targeted autosome loss in aneuploid ES cells, as well as cancer cells.

### Off-target effects of CRISPR/Cas9-mediated chromosome elimination

**Fig. 9:**
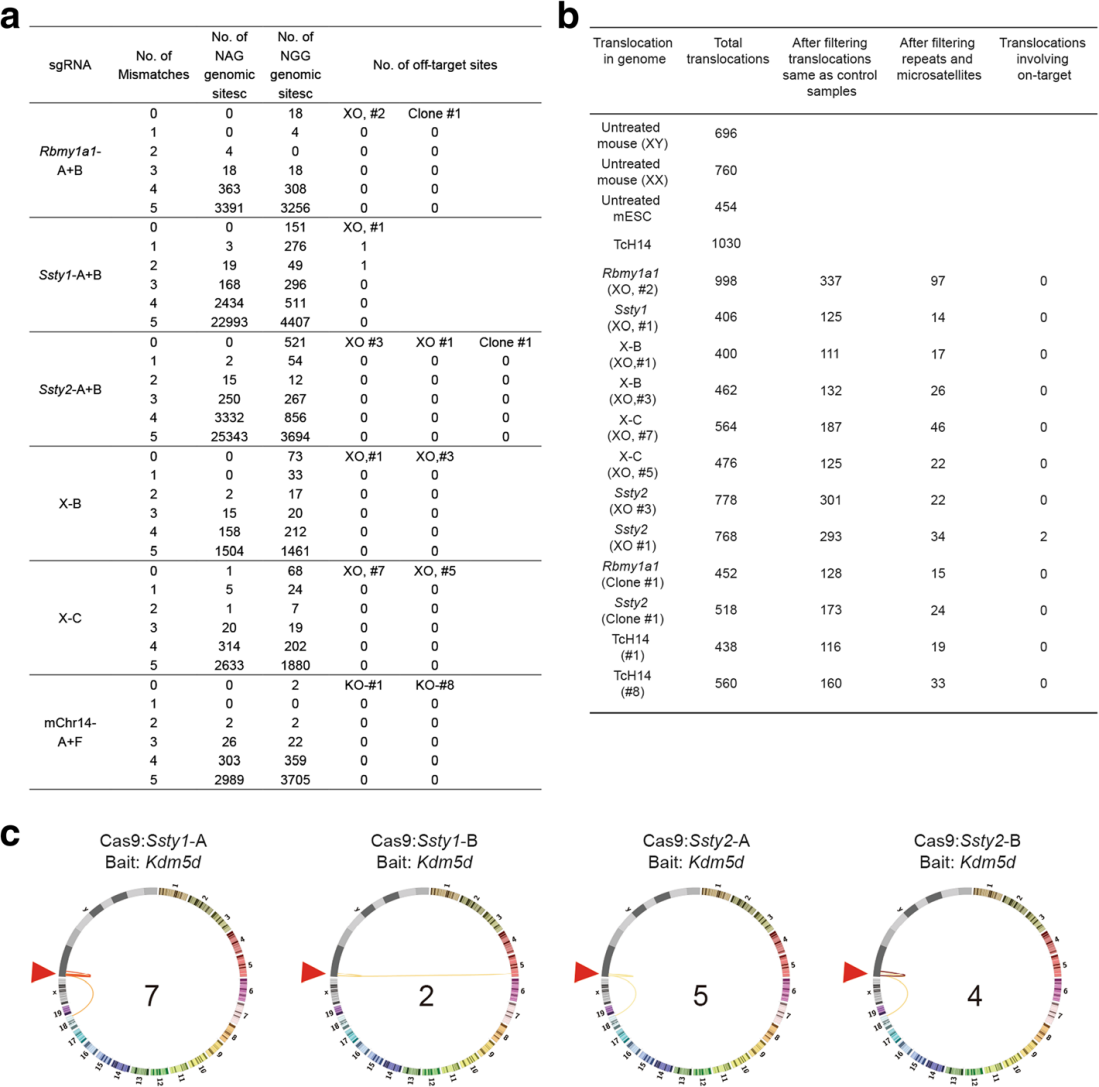
Off-target analysis in cells and animals with chromosome elimination. **a** Summary of indels detected by WGS. See Additional file 1: Table S3 for corresponding mice and cell lines. **b** Summary of genomic rearrangements on the target sequences detected by WGS. **c** Circos plots showing identified off-target hotspots. *Lines* link bait site (*Kdm5d*) to identified off-target hotspots of indicated CRISPR/Cas9 with a range of color from *white* to *dark red*. The higher the frequency, the darker the color. The number of identified off-targets for each CRISPR/Cas9 is shown in the middle of the circus plot. *Arrowheads* indicate the bait CRISPR/Cas9 *Kdm5d* site, but note that the bait CRISPR/Cas9 *Kdm5d* has no detected off-target sites. Three repeats for each treatment (Additional file 1: Table S6 and S7)

We next examined whether CRISPR/Cas9-mediated chromosome elimination induces off-target effects in cells and animals. We first analyzed the off-target effects for each sgRNA in seven to nine female mice obtained by Y chromosome elimination (see Additional file 1: Table S3 for corresponding mice). DNA sequencing of PCR products amplified from these genomic sites showed that mutation rarely occurred at all these loci except *Ssty1*-A (Additional file 1: Figure S5a). We next analyzed off-target sites with up to five mismatches based on WGS described above, including eight mice with Y or X chromosome deletion, and four cell lines with Y chromosome deletion or hChr14 deletion. Among 2186 to 26,469 potential off-target sites for each sgRNA, we found only two off-target sites in only one XO mouse (*Ssty1* #1) after several filtering steps as described in previous studies, including ENSEMBL repeats and microsatellites, variation observed in both mutants and controls (Fig. 9a; Additional file 3: Table S5) [22, 23]. The rest of the mice and cell lines contained no off-target mutations (Fig. 9a; Additional file 3: Table S5). We also examined genomic rearrangements, including deletions, duplications, inversions, and copy number variations, using a strategy described in previous reports [24, 25] and found no rearrangements on the on-target DNA sequence and only two rearrangements in only one XO mouse (*Ssty2* #1) in the predicted off-targeted sites (Fig. 9b; Additional file 3: Table S5). Furthermore, we examined over 100 metaphase FISH samples among 16 XO mice. We observed that all cells showed XO karyotypes with 39 chromosomes, and none showed fluorescent signals of the Y chromosome probe, suggesting no obvious ectopic translocation of Y chromosome fragments to other chromosomes (Additional file 3: Figure S6).

Given that the sample sizes of examined cell clones or mice by WGS and FISH were small and the results cannot reveal rare off-target effects in a population of cells or organisms, we employed high-throughput genome-wide translocation sequencing (HTGTS) [26] to assess off-target activities of the CRISRP/Cas9 systems. To improve the sensitivity of HTGTS, we introduced magnetic bead-mediated DNA recovery after linear amplification to remove extra biotinylated primers and the final libraries were subjected to Hiseq sequencing (see “Methods” for details). Using CRISRP/Cas9 cutting sites at the *Kdm5d* locus as the improved HTGTS (iHTGTS) bait, we barely detected any off-target hotspots for CRISRP/Cas9 targeting the *Kdm5d* site, but did identify several off-target sites for CRISRP/Cas9 targeting the *Ssty1* or *Ssty2* locus (Fig. 9c; Additional file 1: Tables S6 and S7). The off-target sites located in autosomes were identical to those from the WGS results. Additionally, the majority of the determined off-target hotspots were located in the Y chromosome, which was invisible to WGS and FISH in Y chromosome-deleted cells. These Y chromosome-containing off-target sites might further promote Y deletion during chromosome elimination mediated by CRISRP/Cas9, but note that the strongest hotspots harbored only one or two mutation sites in the Cas9-recognition sequences, which should be easily located by bioinformatic prediction. Therefore, strong off-target sites, especially the ones in the same chromosome, should be taken into account during chromosome-elimination using CRISRP/Cas9.

Together, these results indicate that CRISPR/Cas9-mediated chromosome elimination did not induce significant off-target alteration in chromosome-deleted mice and cell lines beyond that expected for CRISPR/Cas9-mediated editing in general [5, 25, 27, 28].

### Mechanism of CRISPR/Cas9-mediated targeted chromosome elimination

**Fig. 10:**
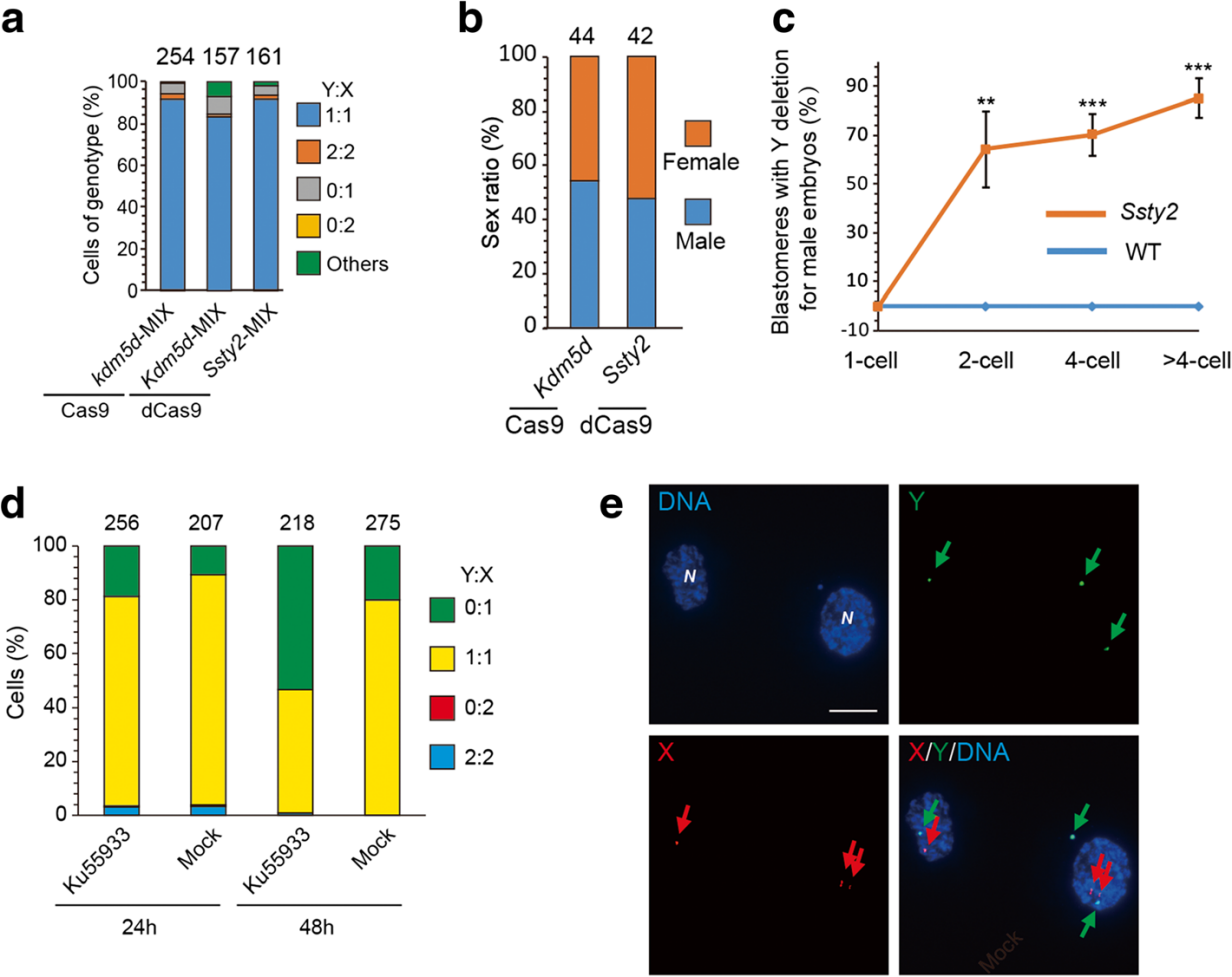
Mechanism of CRISPR/Cas9-mediated targeted chromosome elimination. **a** Results of DNA-FISH analysis on the gene-edited ES cells. Percentages of cells (including dividing cell, Y:X = 2:2 or 0:2) exhibiting different genotype ratios. *n* is the sample size of counted cells. **b** Sex ratio of mice generated by gene editing. *n* is the sample size of mice generated by gene editing. **c** Results of DNA-FISH analysis of the ratio of blastomeres with Y deletion in each male embryo with *Ssty2* targeting at different stages. Data are presented as means ± SEM (n = 4 to 12; ***P < 0.001, **P < 0.01, *P < 0.05, *t* test). **d** Results of DNA-FISH analysis on cells treated with KU-55933 or not. Percentages of cells (including dividing cells, Y:X = 2:2 or 0:2) exhibiting different genotype ratios. *n* is the sample size of counted cells. **e** Examples of Y chromosome exclusion from nucleus after gene editing. DNA-FISH analysis of mouse ES cells or two-cell embryos with isolated Y chromosome following *Ssty2* targeting. *Green*, Y; *red*, X; *blue*, DNA; *N*, primary nucleus*; Arrows: green arrows*, Y; *red arrows*, X. *Bar*, 20 μm

Finally, we continued to explore the molecular mechanism underlying CRISPR/Cas9-mediated targeted chromosome elimination. We first checked whether multiple DNA cleavages on the targeted chromosome and cell division are necessary for chromosome elimination. We treated mouse ES cells or embryos with Cas9 and sgRNA targeting *Kdm5d* (only one copy on Y chromosome) or dCas9 and sgRNA targeting *Ssty2* and found no Y chromosome elimination (Fig. 10a, b). To monitor the process of Y chromosome elimination, we stained the injected embryos at different stages from the one- to eight-cell stage. We found no Y chromosome elimination in one-cell embryos, harvested at 6 h after sgRNA injection (Fig. 10c). However, Y chromosome elimination was observed at the two-cell stage (65%) and increased further at the four- to eight-cell stages (85%) (Fig. 10c). We also tested whether impairing DNA repair by ATM inhibitor KU-55933 could increase the efficiency of chromosome elimination. Mouse ES cells were transfected with Cas9 and sgRNA targeting *Ssty2* and then treated with KU-55933. We found that cells treated with KU-55933 for 48 h could increase Y chromosome elimination efficiency by 2.65-fold (Fig. 10d). These results indicate that multiple DNA cleavages on the targeted chromosome, cell division, and DNA repair efficiency are necessary for chromosome elimination.

We next examined whether multiple CRISPR/Cas9-mediated cleavages on a targeted chromosome produce micronuclei, resulting from pulverization of chromosomes [29]. After cells were treated with *Ssty2* sgRNA, we found that micronuclei-containing Y chromosome was observed around the primary nucleus of cells (Fig. 10e). This chromosome loss may be caused by nuclear exclusion of the targeted chromosome followed by cytoplasmic degradation, a process that requires further study.

## Discussion

A very recent study [30] reported that the Y chromosome could be deleted in ES cells and zygotes by CRISPR/Cas9-mediated genome editing. Here we have achieved complete elimination of the Y chromosome by multiple CRISPR/Cas9-mediated DNA cleavages on the targeted chromosome in ES cells, cells in vivo, and zygotes with high efficiency. Notably, using this approach to eliminate the X chromosome in mouse embryos with the XX karyotype, one of two homologous X chromosomes could be efficiently deleted. However, the remaining X chromosome was also mutated, with indels or fragment deletion in the targeted region. Most chromosome-specific repeated sequences are located in non-coding regions, and thus we could minimize these side effects by targeting the non-coding DNA sequences within small regions (< 2 kb) without obvious biological functions. Alternatively, in principle, these indels and large deletions could be avoided by sgRNAs that target only one of the two homologous chromosomes, based on single nucleotide polymorphism. As shown in Fig. 5, we could delete the Y chromosome with 14 single-target sgRNAs. Nevertheless, reducing the number of sgRNAs and improving the efficiency of chromosome elimination may make this approach more applicable.

We have shown that multiple CRISPR/Cas9-induced DNA cleavages could promote extra hChr14 or hChr21 loss in aneuploid ES cells, as well as hChr7 in human cancer cells. However, we failed to obtain aneuploid mouse ES cells or embryos with autosome deletion (data not shown). We surmise that single-autosome deletion would inhibit cell growth or lead to embryonic lethality. Thus, using aneuploid cells with trisomy autosomes, such as cells from DS patients containing three hChr21s, we detected autosome elimination by CRISPR/Cas9 editing.

Recently, Yang et al. [31] reported that CRISPR/Cas9-mediated editing of porcine endogenous retroviruses (PERVs) could remove repetitive sequences (up to 62 copies) but did not delete chromosomes. In comparison with this study, our strategy to eliminate chromosomes is to use sgRNAs targeting repeat sequences in a single chromosome, rather than repeat sequences scattered in many chromosomes. Consistent with this study [31], we observed no obvious off-target mutations or chromosome rearrangements in all examined mouse ES cells and mice with chromosome elimination. Notably, we observed that multiple CRISRP/Cas9-mediated DNA cleavages could produce partial deletion of the targeted chromosome in mice, aneuploid mouse ES cells, and cancer cells, as well as chromosome rearrangement in cancer cells. Furthermore, several off-target sites for CRISRP/Cas9 targeting the *Ssty1* or *Ssty2* locus were detected by both genome-wide off-target assay (iHTGTS) and independent WGS analysis. Therefore, the evaluation of off-target effects by both in silico and in vivo approaches should be taken into account when designing CRISRP/Cas9 systems for chromosome elimination and may be needed before this approach can be used clinically without risk.

Although there are many mouse models for aneuploidy diseases, such as the XO mouse for Turner syndrome, many features of patients with Turner syndrome could not be well replicated in those mouse models, including the two most common—short stature and premature ovarian failure—which affect over 90% of recognized patients [15, 18]. CRISPR/Cas9-mediated targeted chromosome elimination dramatically transforms our ability to generate disease models in diverse organisms, such as in non-human primates. Moreover, this approach would provide a potential therapeutic approach to cure aneuploidy diseases, including DS, Klinefelter syndrome, and XYY syndrome [3, 4, 21, 32–36].

Aneuploidy is a hallmark of cancer [37], and although it can impair cell proliferation and change cell metabolism, it could also promote cell growth under selective pressure, in which context it might contribute to tumorigenesis [38, 39]. Compounds such as AICAR, chloroquine, and 17-AAG, which cause lethality only in aneuploid cells, are in clinical trials of their antitumor activity in multiple myeloma and anaplastic large cell lymphoma [40]. CRISPR/Cas9-mediated targeted chromosome elimination offers a new approach for studying aneuploidy in tumorigenesis and a potential treatment strategy against a broad spectrum of human tumors.

To our knowledge, this is the first report on X and autosome chromosome elimination via genome editing [41–43]. It paves the way for a potential genetic approach to chromosome therapy in vivo.

## Methods

### Production of Cas9 mRNA and sgRNA

Bicistronic expression vector px330 expressing Cas9, mCherry, and sgRNA was digested with BbsI, and the linearized vector was purified using the Universal DNA Purification Kit (Tiangen). A pair of oligos (Additional file 1: Table S8) for each targeting site was annealed, phosphorylated, and ligated to the linearized vector. The T7 promoter was added to the Cas9 coding region by PCR amplification of px260, using primer Cas9 F and R (Additional file 1: Table S8). The T7-Cas9 PCR product was purified using the Universal DNA Purification Kit (Tiangen) and used as the template for in vitro transcription (IVT) using mMESSAGE mMACHINE T7 ULTRA kit (Life Technologies). The T7 promoter was added to the sgRNA template by PCR amplification of px330, using primers listed in Additional file 1: Table S8. The T7-sgRNA PCR product was purified and used as the template for IVT using a MEGA shortscript T7 kit (Life Technologies). Both the Cas9 mRNA and the sgRNAs were purified using a MEGAclear kit (Life Technologies) and eluted in RNase-free water.

### One-cell embryo injection

All animal procedures were performed under the ethical guidelines of the Institute of Neuroscience, Chinese Academy of Sciences. C57BL/6 J or B6D2F1 female mice and ICR mouse strains were used as embryo donors and foster mothers, respectively. Super-ovulated female C57BL/6 J (4 weeks old) or B6D2F1 mice (7–8 weeks old) were mated to C57BL/6 J or B6D2F1 stud males, and fertilized embryos were collected from the oviduct. Cas9 mRNA (50 ng/μl) and sgRNA (50 ng/μl for each sgRNA in single to three sgRNA injections, 20 ng/μl for each sgRNA in the 14-sgRNA cocktail injection) were injected into the cytoplasm of fertilized eggs with well-recognized pronuclei in HCZB medium containing 10 μg/ml Cytochalasin B (CB). The injected zygotes were cultured in KSOM with amino acids at 37 °C under 5% CO_2_ in air until the two-cell stage by day 1 or blastocyst stage by day 3.5. Thereafter, 20 two-cell embryos were transferred into the oviduct of pseudo-pregnant ICR females at 0.5 dpc. The blastocysts were used for deriving ES cells.

### Karyotype analysis

Fibroblasts, bone marrow cells, or ES cells were used for karyotyping. Fibroblasts were derived from mouse tails, which were cut into small pieces and cultured for 7 days. Then fibroblasts or mouse ES cells were incubated with 200 ng/ml demecolcine (Sigma) for 1 h. For bone marrow cells, mice were injected with 15–20 μg demecolcine per mouse and bone marrow cells were isolated 4 h later. The fibroblasts, bone marrow cells, or ES cells were re-suspended in 0.075 M KCl at 37 °C for 10–30 min, followed by carnoy’s fixative (25% acetic acid in methanol) for 30 min cell plating on pre-cleaned slides. For chromosome number counting, the slides were stained with Hoechst 33342. For G banding, the slides were incubated with 0.025% pepsin and then stained with Giemsa for 15 min. More than ten metaphase spreads were analyzed.

### DNA-FISH

Fibroblasts, mouse ES cells, bone marrow cells, or HT-29 cells were harvested, incubated in 0.075 M KCl, and then fixed in 3:1 methanol:glacial acetic acid (v/v) at 4 °C, and dropped onto microscope slides. For embryos, the zona pellucida was removed with Tyrode’s acid and collected onto slides after fixation. The slides were aged at 37 °C overnight, dehydrated through an ethanol series (70, 90, and 100% ethanol for 5 min each) at room temperature and air-dried, and then denatured in 70% formamide/2× SSC at 75 °C for 5 min followed by immediate hydration in a −20 °C precooled ethanol series (100, 90, and 70%). The probe (Additional file 4: Table S9, listed below) was denatured in a water bath at 75 °C for 5 min. The slides were hybridized in a humidified chamber overnight at 37 °C and rinsed 2 × 5 min in 50% formamide/2× SSC at 42 °C, 2 × 5 min in 2 × SSC at 42 °C the following day. Finally, the slides were stained with 10 μL DAPI-antifade solution and mounted with a coverslip. The samples were captured using an Olympus BX53 fluorescent microscope or Nikon Nie-A1 plus fluorescent microscope. To count probe spots in metaphase spreads, an image of DAPI and a merged image of DAPI and probe signal were analyzed together. For counting probe spots in interphase spreads, spots were counted by two individuals.

### Derivation of ES cells

Morulae or blastocysts were selected to generate ES cell lines. The zona pellucida was removed using acid Tyrode solution. Each embryo was transferred into one well of a 96-well plate seeded with embryonic fibroblast feeders in ES cell medium supplemented with 20% knockout serum replacement, 1500 U/ml leukemia inhibitory factor (LIF), 3 μM CHIR99021, and 1 μM PD0325901. After 4–5 days in culture, the colonies were trypsinized and transferred to a 96-well plate with a feeder layer in the fresh medium. Clonal expansion of the ES cells proceeded from 48-well plates to six-well plates with feeder cells and then to six-well plates for routine culture.

### Derivation of aneuploid ES cells

Mitotic donor cells were obtained after culturing cells with colcemid (75 ng/mL) 10–12 h at 37 °C. Cells were sedimented at 1000 rpm for 10 min and resuspended in 10 ml of chromosome isolation GH buffer. Cells were incubated at 37 °C in a water bath for 10 min and then on ice for 5 min. We added 100 μl of 10% Triton X-100 to cells (final concentration is 0.1%) and incubated them on ice for 5 min. Cells were then lysed by passing three times through a 23G needle. The homogenate was centrifuged at 1000 rpm for 10 min. Supernatant was collected into a new tube and chromosomes were spun down at 2500 rpm for 20 min. Chromosomes were resuspended in 1 ml of HCZB. Zygotes were obtained from the oviducts of superovulated female mice after mating. The chromosomes were microinjected into denuded zygotes using a piezo-driven micropipette 3–4 μm in diameter. Injected zygotes were cultured in vitro until 3.5 dpc in KSOM (aa). Details of the derivation of mouse ES cells are described in the “Derivation of ES cells” section.

### Cell culture and transfection

129/Sv × C57BL/6 ES cells were cultured on feeder cells using standard ES cell culture conditions. Cells were transfected with px330 expressing Cas9, mCherry, and sgRNA using Lipofectamine 3000 Reagent (Invitrogen) according to the manufacturer’s instructions. Forty-eight hours after transfection, mCherry-positive ES cells were sorted into 96 wells using BD FACS AriaII for further culturing. After 7 days of culturing, the colonies were picked up and expanded for further analysis.

For cell treatment with drugs, the ATM inhibitor KU-55933 (number S1092, Selleckchem) was used at 20 μM. Cells were transfected with plasmids (pX330-mCherry-*Ssty2*-A and B) and mCherry-positive mouse ES cells were sorted by FACS 12 h after transfection. DNA-FISH analysis was performed 24 or 48 h later.

Human iPSCs were purchased from ATCC (ATCC® ACS-1003™) and cultured on irradiated mouse embryonic fibroblast (iMEFs) feeder layers in serum-free N2B27-LCDM medium as described previously [44]. For transfection, cells were dissociated using TrypLE, replated in iMEF-coated 12-well plates, and transfected in suspension with gRNAs, Cas9, and EGFP or mCherry plasmid using Lipofectamine 3000. Twenty-four hours after transfection, EGFP^+^/mCherry^+^ cells were sorted and used for DNA-FISH analysis at 7 days post-transfection.

### WGS and off-target analysis

We firstly screened the whole mouse (mm10) and human (hg19) genome for chromosome-specific sgRNAs with our in-house script (https://github.com/pingjie/findChrCrispr). The sgRNAs for each chromosome given by our software are listed in Additional file 1: Tables S2 and S3.

WGS was carried out using Illumina HiSeq X Ten at mean coverages of 20×. Qualified reads were mapped to the mouse reference genome (mm10) by speedseq [45] (https://github.com/hall-lab/speedseq) with default parameters. FreeBayes (v0.9.10) [46] and LUMPY [24] (https://github.com/arq5x/lumpy-sv) were run on the aligned sequence files (BAM files) for short indel detection and structural variation discovery. We firstly filtered germ-line variants which were the same as variants in the “control” (wild-type) samples (untreated mice-XY, untreated mice-XX, untreated mESCs, and TcH14). These results were then filtered to remove variation which overlapped any UCSC repeat regions and microsatellite sequences. The original bam files (pileup) around each candidate variation site were further examined to eliminate those cases where potentially shared variation with “control” samples were not detected by the variant calling software. Next, the raw variant output was manually inspected to remove variants which overlapped with any of the four wild-type samples. For the short indel variations, homopolymers with unit length greater than 2 bp were also removed [22]. Variations after each filtering step are listed in Additional file 1: Table S5.

To search for rearrangements involving on-target DNA sequences that might have integrated elsewhere in the genome, we detected whole genome translocation cases [24, 25] in each sample. After filtering translocations in both mutant and wild-type samples that overlapped with the UCSC repeat regions and microsatellites, the translocations involving the on-target sequences were not observed in any of our mutant samples.

Potential off-target sites of sgRNAs were predicted as previously reported [47] (http://www.rgenome.net/cas-offinder/). We extracted all the off-target sites with no more than five mismatches for each sgRNA. We searched the short indel variations within the 23-bp predicted off-target sites, and structural variations within 250 bp up- or downstream of the potential off-target sites (Additional file 3: Table S5).

### iHTGTS

Mouse ES cells were transfected with plasmids carrying indicated CRISPR/Cas9 and were harvested 48 h later and then digested with Protease K to extract for genomic DNA. The iHTGTS libraries were prepared following the protocol described previously with minor modifications [26]. Briefly, linear amplification was performed with 20 nM biotinylated primer (biotin-CCCATTTGCTATTGTTGACAGGAAACCCACTGCC, by Sangon, Shanghai) for 80 cycles; extra primers were removed by 1.2× AxyPrep Mag PCR Clean-Up beads (Axygen, US). Locus-specific primer CTTTGGAGTGAATGTCTGCTCC was used for nested PCR. *Kpn*I was used to block germline fragments, and 1.0× AxyPrep Mag PCR Clean-Up beads (Axygen, US) were used to recover the DNA after enzyme blocking. All the iHTGTS libraries were sequenced by Hiseq. Bioinformatic analysis for off-targets followed the protocol described previously [48].

### Genotyping

Genotyping of the mice was performed by PCR using DNA extracted from their tails. Single ES cell clones were genotyped by nested PCR. The single clone was dissolved in DNA lysis buffer (4 μg/μl proteinase K, 0.1% Triton X 100, and 0.1% Tween 20 in nuclease-free water). The samples were digested at 55 °C for 30 min and then the proteinase K was inactivated at 95 °C for 5 min. PCR was performed using specific primers (Additional file 1: Table S8) under the following conditions: 95 °C for 5 min followed by 35 cycles of PCR (95 °C for 30 s, 58 °C for 30s, and 72 °C for 120 s) for mouse. The nested PCR was 95 °C for 5 min followed by 25 cycles of PCR (95 °C for 30 s, 62 °C for 30s, and 72 °C for 90s) for mouse ES cells. Secondary PCR was performed using 0.5 μg primary PCR product and nested inner primer. PCR was carried out with the same reaction mixture and cycle parameters.

### Plasmid DNA delivery into mouse embryos by in utero electroporation

In utero electroporation (IUE) experiments were performed using ICR mice. IUE was performed as previously described [49]. Briefly, E14.5 pregnant ICR mice were anesthetized with sodium pentobarbitone (50 mg/kg), and the uterine horns were exposed. Plasmid mixture (1 μL; containing the px330-EF1α-EGFP-*Ssty2*-A sgRNA (1 μg/μl), px330-EF1α-EGFP-*Ssty2*-B sgRNA (1 μg/μl)) with 0.01% Fast Green dye (Sigma)) was injected into the embryos’ lateral ventricle with a glass micropipette. For electroporation, five pulses with a 50 ms duration separated by 950 ms were applied at 32 V using ECM 830 (BTX). Then the uterine horns were placed back into the abdominal cavity to allow the embryos to continue normal development. Forty-eight hours after IUE, the embryos were collected and dissected for FACS.

## Conclusion

In summary, we demonstrated that a single specific chromosome, including a sex chromosome and an autosome, could be selectively eliminated via CRISPR-mediated multiple DNA cleavages on the targeted chromosome in culture cells, embryos and in vivo tissues. With the increase of efficiency and specificity, we believe CRISPR/Cas9-Mediated targeted chromosome elimination would be broadly applicable in developing animal models and therapeutic treatments for aneuploidy.

## Additional file

Additional file 1: Supplemental tables and figures. (DOCX 3253 kb)
Additional file 2: Table S2. Unique and multiple repeated sequences for targeting by a single specific sgRNA. (XLSX 9524 kb)
Additional file 3: Table S5. Summary of genomic variations and filtering steps. (XLSX 19 kb)
Additional file 4: Table S9. Probes used in this work. (DOCX 509 kb)
