# Supplementary material for: CRISPR/Cas9-mediated targeted chromosome elimination
Source: Genome Biol. 2017 Nov 24;18:224. doi: 10.1186/s13059-017-1354-4 (PMC5701507; doi:10.1186/s13059-017-1354-4)
Supplement: Additional file 1: — Supplemental tables and figures. (DOCX 3253 kb) [file 13059_2017_1354_MOESM1_ESM.docx]

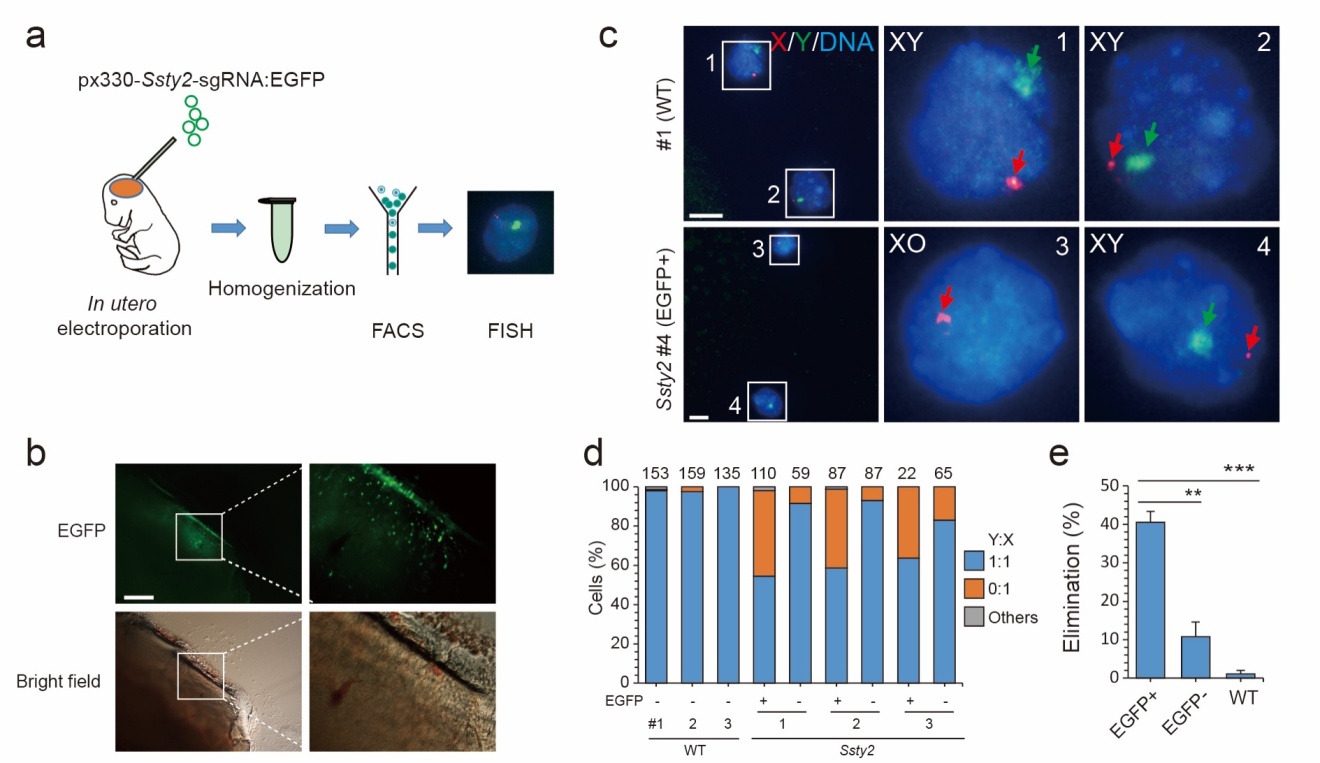


**Figure S1.** CRISPR/Cas9 - mediated Y chromosome elimination *in vivo*. (**a**) Experimental scheme for Y chromosome elimination in fetal brain via *in utero* electroporation. The constructs of Cas9/sgRNA-*Ssty2*-A and Cas9/sgRNA-*Ssty2*-B were delivered into mouse fetal brain at E14.5 via *in utero* electroporation, with EGFP co-expression. Two days later, electroporated tissue were dissected and cells were sorted by FACS for DNA FISH analysis. (**b**) Images of cortex of the embryo at E16.5 after electroporation. EGFP, transfected cells. Bar, 500 μm. (**c**) Representative DNA-FISH analysis of transfected cells with Y chromosome editing. Green: Y probe; red: X probe for XqC3; blue: DNA. Arrows: green, Y; red, X. Numbered squares: single cells shown at a higher resolution on the right panel. Bar, 20 μm. (**d**) Stacked bar graphs showing results of DNA-FISH analysis on the electroporated cells from mouse fetal brain at E16.5. Percentages of cells exhibiting different ratio of genotype. ‘n’: sample size of cells counted. Results were obtained from 3 wild-type mice and 3 electroporated mice. (**e**) Efficiency of Y chromosome elimination in EGFP^+^ (*Ssyt2*-positive cells), EGFP^-^ (*Ssyt2*-negative cells) and unelectroporated cells, based on DNA-FISH analysis (n = 3, ***P < 0.001, **P < 0.01, *t* test).


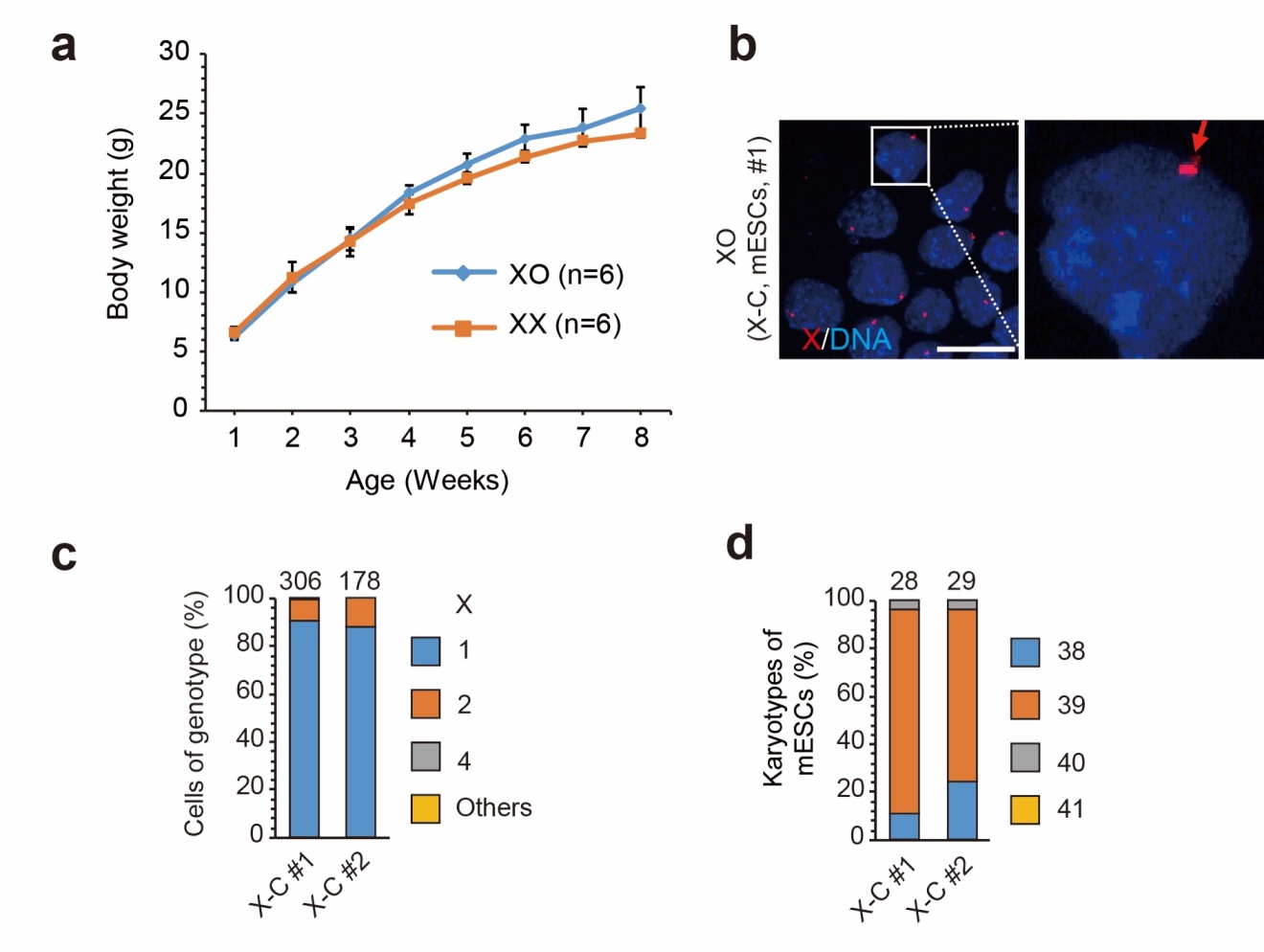


**Figure S2.** Analysis of XO mice and mouse ES cells generated from gene-edited embryos for X chromosome elimination. (**a**) The weight of XO mice (X-B, n=3; X-C, n=3) and the siblings of XX mice (n=6) were measured about once a week from 1 weeks to 9 weeks. Means ± SEM. (**b**) DNA-FISH analysis mESCs of the X-C XO #1. The pure XO ES cells show the deletion of one copy of X chromosome. Red: X chromosome probe for XqA7.3; Blue: DNA; Arrows: X chromosome. (**c**) Stacked bar graphs showing percentage of pure XO ES cells derived from gene-edited embryos with different karyotypes. Related to Figure 6. (**d**) Stacked bar graphs showing percentage of different karyotypes in mouse ES cells (X-C #1, X-C #2). ‘n’: sample size counted.


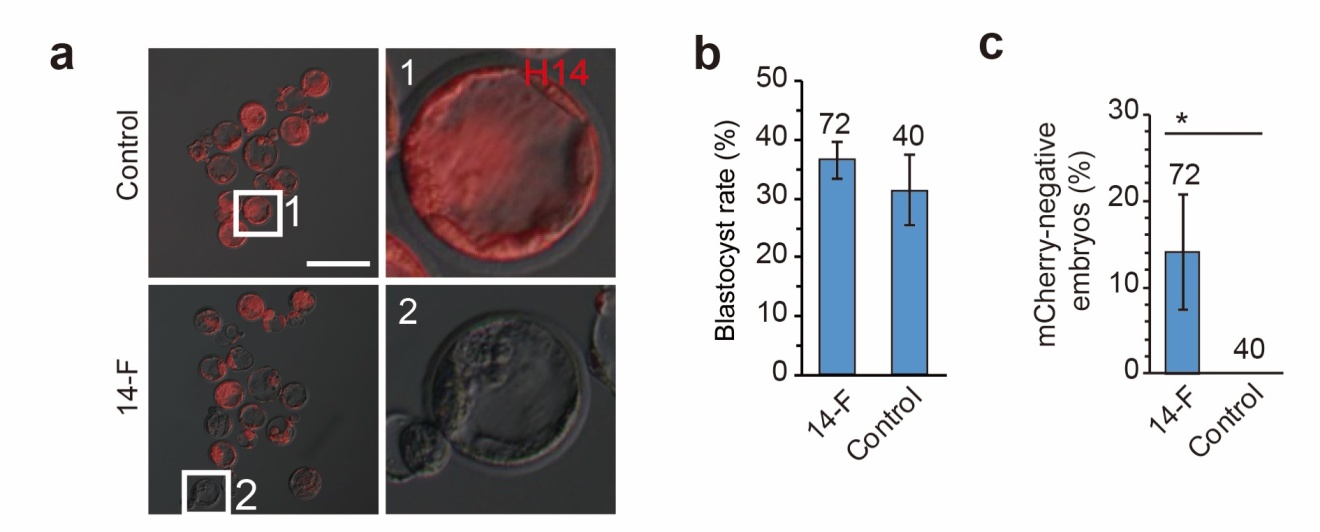


**Figure S3.** Elimination of hChr14 in reconstructed embyros. (**a**) Representative immunofluorescence images of gene-edited embryos at blastocyst stage. Single TcH14 cell was injected into mouse oocyte and the reconstructed embryo was then injected with Cas9 mRNA and sgRNA-14-F 6 hours later. The reconstructed embryos were cultured into blastocysts and observed at microscope. Square, blastocysts shown at a higher resolution on the right panel. (**b**) Blastocyst rate of reconstructed embryos with gene editing. ‘n’: sample size of injected embryos.


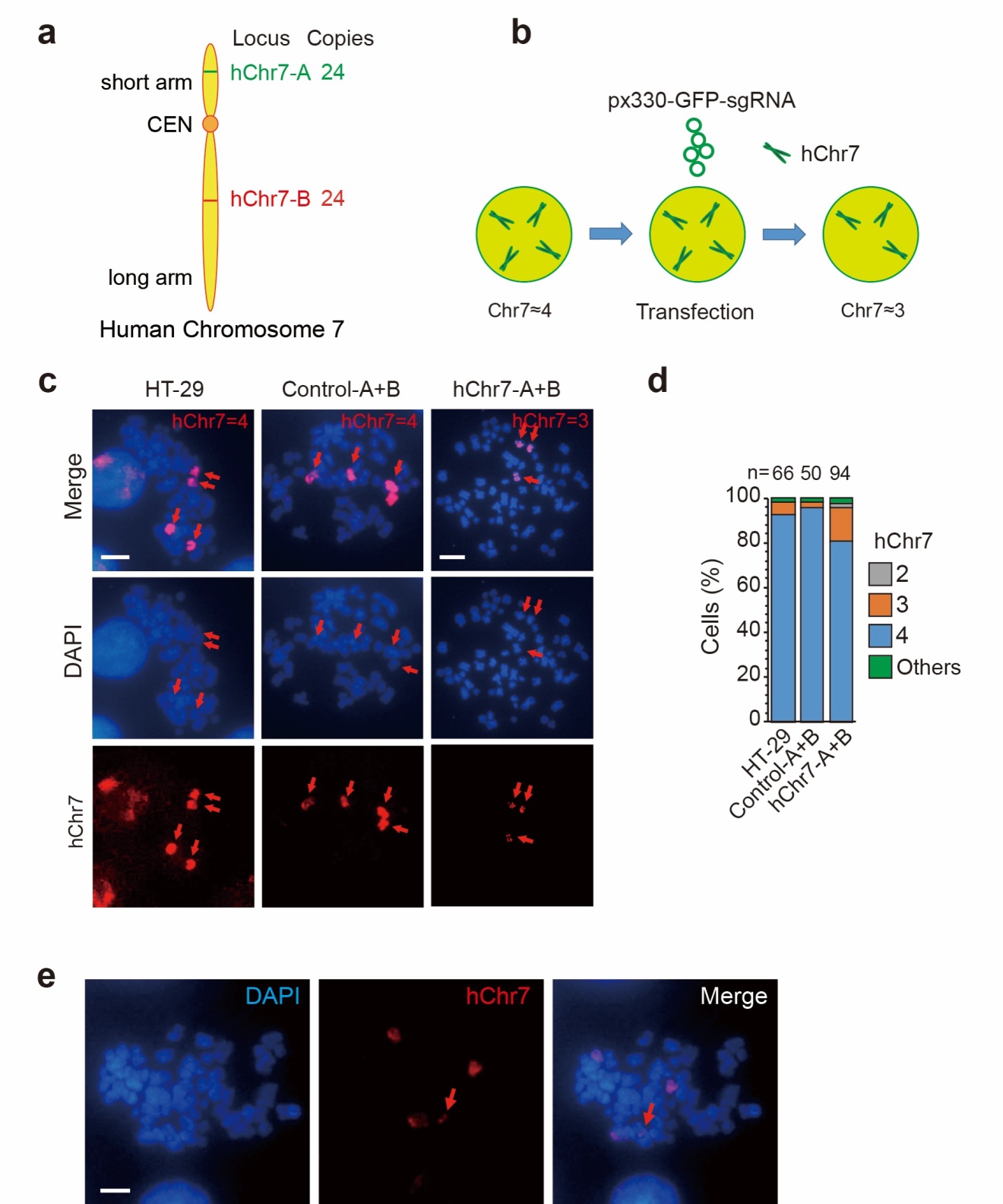


**Figure S4.** Elimination of human chromosome 7 in cancer cells by CRISPR/Cas9-mediated gene editing. (**a**) Schema of targeted gene loci in hChr7. (**b**) Experimental design. HT-29 cells were transfected with plasmids expressing Cas9, chromosome targeting sgRNAs, and mCherry. One day later, GFP-positive cells were FACS-sorted and cultured on 6 wells for DNA-FISH analysis. (**c**) Representative DNA-FISH analysis of transfected HT-29 cells with hChr7-A +B or control sgRNAs. Red: Texas red-labeled whole-chromosome probe for hChr7; Blue: Hoechst 33342-labeled DNA; Arrows: hChr7. Numbered square: single cells shown at a higher resolution on the right panels. Bar, 20 μm. (**d**) Stacked bar graphs showing results of DNA-FISH analysis on the metaphase gene-edited ES cells. Percentages of metaphase cells exhibiting different ratio of genotype. ‘n’: sample size of metaphase cells counted. (**e**) Example of chromosome rearrangement occurred in transfected HT-29 cells with hChr7-A+B. Red: Texas red-labeled whole-chromosome probe for hChr7; Blue: Hoechst 33342-labeled DNA; Arrow: hChr7 fragment. Bar, 20 μm.

**
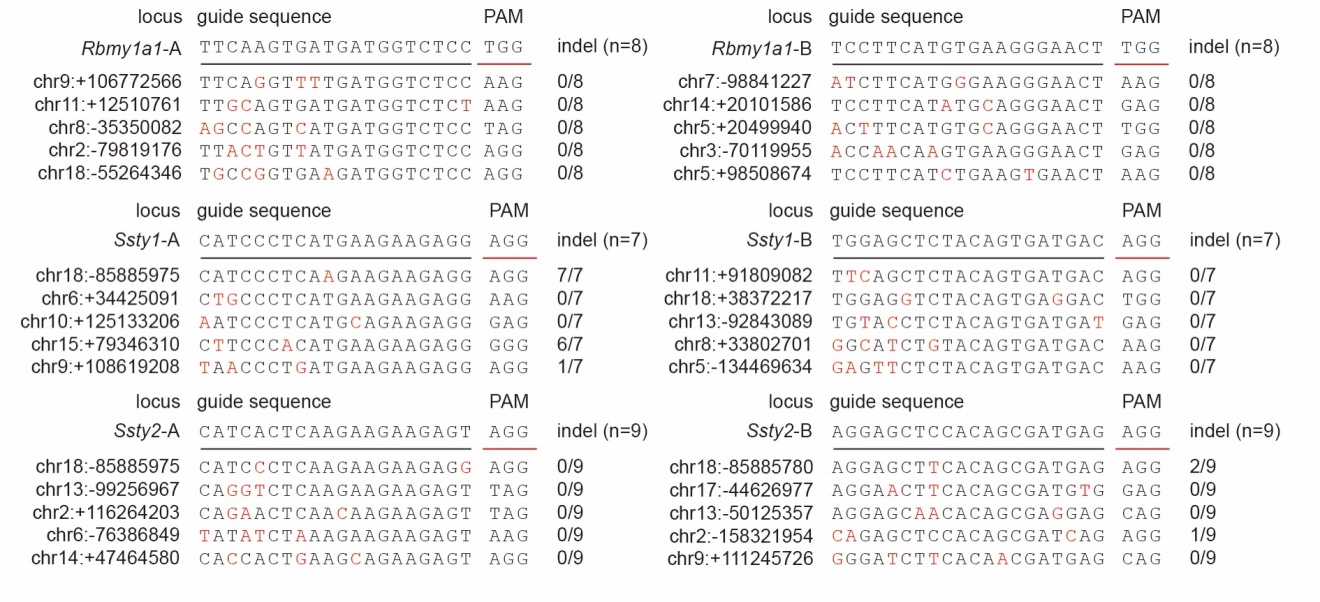
**

**Figure S5.** Off-target analysis in animals with chromosome elimination. We analyzed off-target effects for each sgRNA in 7 to 9 female mice from Y chromosome editing, listed in Table S3. We used the tool developed by Hsu et al. for predicting off-target loci. Top 5 potential off-target sites were selected for analysis (Table S3). DNA sequencing of PCR products amplified from these genomic sites showed that very few mutations had occurred at *Rbmy1a1*-A, *Rbmy1a1*-B, *Ssty1*-B, *Ssty2*-A and *Ssty2*-B. For *Ssty1*-A, three loci showed indels. Red: mismatch with targeted sequence.

**
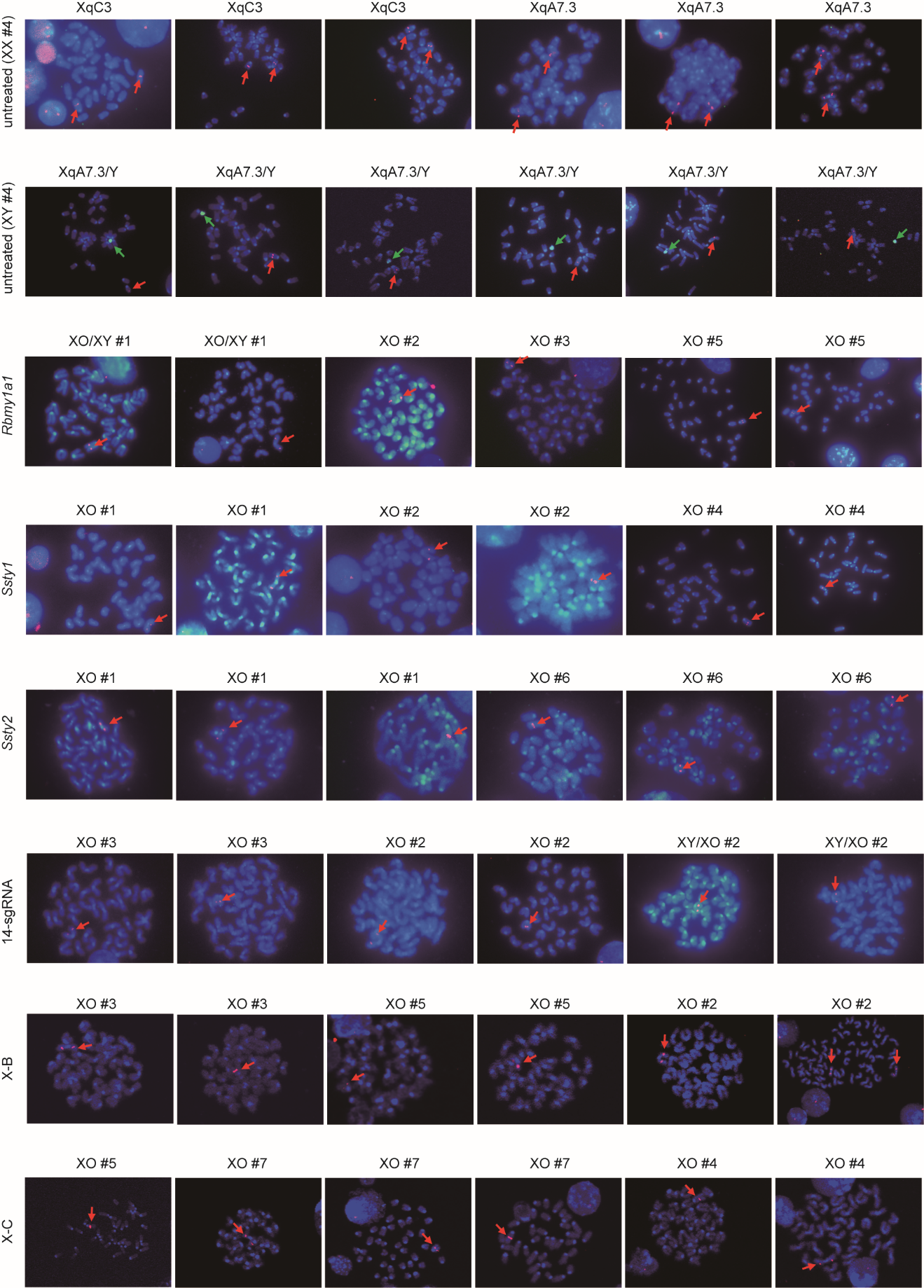
**

**Figure S6.** Metaphase FISH of the gene-edited mice. Tail tissues or bone marrows of the gene-edited mice (*Rbmy1a1, Ssty1, Ssty2*, 14-sgRNA cocktail, X-B, X-C) and control mice (untreated XY, untreated XX) were performed DNA-FISH and metaphase FISH were analyzed. Green: whole chromosome probe for Y chromosome; Red: X chromosome probe for XqA7.3 or XqC3; Blue: DNA.

**Table S1. sgRNA target sequence used for chromosome elimination.**

| Chromosome | Locus | Sequence | Copies |
| --- | --- | --- | --- |
|  |  |  |  |
| Mouse ChrY | *Rbmy1a1* | A: TTCAAGTGATGATGGTCTCCTGG | >50 ^1^ |
|  |  | B: TCCTTCATGTGAAGGGAACTTGG | >50 ^1^ |
|  | *Ssty1* | A: CATCCCTCATGAAGAAGAGGAGG | >35 ^1^ |
|  |  | B: TGGAGCTCTACAGTGATGACAGG | >35 ^1^ |
|  | *Ssty2* | A: CATCACTCAAGAAGAAGAGTAGG | >30 ^1^ |
|  |  | B: AGGAGCTCCACAGCGATGAGAGG | >30 ^1^ |
|  | *Kdm5d* | A: GATGGTACCTACAGAAGTTGTGG | 1 |
|  |  | B: GGACTTATCTCCTGAAGAAAAGG | 1 |
|  | *Eif2s3y* | A: ACAATTGGTCATGTTGCTCATGG | 1 |
|  |  | B: AGGAATATTACCATAAAACTTGG | 1 |
|  | *Zfy1* | A: GGATAGTGACCAGATTGTTGTGG | 1 |
|  |  | B: CCTGAGCAAGTTCTCAATTTAGG | 1 |
|  | *Ube1y1* | A: GAAACCCCTACTTGCCTCGCTGG | 1 |
|  |  | B: CTCAGCACAGCAGGCCTCCTCGG | 1 |
|  | *Ddx3y* | A: GTCTGTGATAAGGACAGTTCAGG | 1 |
|  |  | B: GTGATCGTGGAAGTGGATCCAGG | 1 |
|  | *Usp9y* | A: ACTCTGGCTCTGTGTTTCCCAGG | 1 |
|  |  | B: AGGATTAGACTACCTTTGGAAGG | 1 |
|  | *Zfy2* | A: ATAATCCTCACAGTTGATTCTGG | 1 |
|  |  | B: CAATGAGGTTGATGTAATACTGG | 1 |
| Mouse ChrX | X-A | CTGGCTTCCTTACCCACTGCTGG | 128 |
|  | X-B | GCTTGGTTAGGGTGAGTGCTTGG | 72 |
|  | X-C | CTGGGAATTAGAATGCCAGAAGG | 66 |
|  | X-D | TAAGTGCTGTGTGCTGCTACTGG | 44 |
|  | X-E | TGTCATGACCTCTCTGTGACTGG | 50 |
| Mouse Autosome | *Tyr*-A | GGGAAGGGTTACTCAGAGTCAGG | 1 |
|  | *Tyr*-B | CCAGAAGCCAATGCACCTATCGG | 1 |
|  | *Tyr*-C | TACAGCTACCTCCAAGAGTCAGG | 1 |
| Human Chr14 | HChr14-A | GAGCAACAACCTAGCATCTCAGG | 107 |
|  | HChr14-B | CCACACCACTGTCCGCGTAGTCG | 98 |
|  | HChr14-C | CTCGAAATCCTTGACAATCCAGG | 77 |
|  | HChr14-D | CCTGTGGCACCCTCAGGAGCAAC | 72 |
|  | HChr14-E | ATAGGATGCGAGGTTTAGATAGG | 22 |
|  | HChr14-F | CCATCGCCCCAACCAACTCTTCT | 22 |
| Human Chr21 | HChr21-A | GGAGGCTCGGTGCAGGTAAGGGG | 49 |
|  | HChr21-B | CTGTGAGCATCCTCTGTGGATGG | 24 |
|  | Control-A | GGCACGTACCGTGCCACACAGGG | 1 |
|  | Control-B | GCTCTGGGTCTCTAGAAGAATGG | 1 |
| Human Chr7 | Hchr7-A | ACAGGTCTCCCGGGTGGCACAGG | 24 |
|  | Hchr7-B | AGTGATGTCATCATCCTGCGTGG | 24 |
|  | Control-A | GACAGAAAGAGGCATCCACTGGG | 1 |
|  | Control-B | GGACAAAGAGAGGGGTCCTCGGG | 1 |

**Table S3.** Karyological characteristics of mice and ES cells for Y or X chromosome elimination.

| Locus | Name | Background | Karyotyping | | | | Geno- typing | DNA-FISH | Whole genome sequence | Mating | Off- target |
| --- | --- | --- | --- | --- | --- | --- | --- | --- | --- | --- | --- |
|  |  |  | 38 | 39 | 40 | 41 |  |  |  |  |  |
| *Rbmy1a1* | XO #1 | B6D2F1 | 0 | 11 | 0 | 0 | Y | / | / | / | Y |
| *Rbmy1a1* | XO #2 | B6D2F1 | 0 | 11 | 1 | 0 | Y | Y | Y | / | Y |
| *Rbmy1a1* | XO #3 | B6D2F1 | 1 | 13 | 3 | 0 | Y | Y | / | / | Y |
| *Rbmy1a1* | XO #4 | B6D2F1 | 1 | 20 | 0 | 0 | Y | Y | / | / | Y |
| *Rbmy1a1* | XO #5 | B6D2F1 | 0 | 10 | 2 | 0 | Y | Y | / | / | / |
| *Rbmy1a1* | XO #6 | B6D2F1 | 0 | 11 | 1 | 0 | / | / | / | / | / |
| *Rbmy1a1* | XO #7 | C57BL/6 | 1 | 9 | 1 | 0 | / | / | / | Y | / |
| *Rbmy1a1* | XO #8 | C57BL/6 | 0 | 10 | 1 | 0 | / | / | / | Y | / |
| *Rbmy1a1* | XO/XY #1 | C57BL/6 | 0 | 9 | 6 | 0 | Y | Y | / | Y |  |
| *Rbmy1a1* | XO/XY #2 | C57BL/6 | 1 | 12 | 16 | 0 | Y | / | / | Y | Y |
| *Rbmy1a1* | XO/XY #3 | C57BL/6 | 0 | 12 | 6 | 1 | Y | Y | / | Y | Y |
| *Rbmy1a1* | XO/XY #4 | C57BL/6 | 0 | 8 | 3 | 0 | Y | Y | / | Y | Y |
| *Rbmy1a1* | XX #1 | C57BL/6 | 0 | 1 | 14 | 0 | / | / | / | / | / |
| *Rbmy1a1* | XX #2 | B6D2F1 | 0 | 1 | 8 | 1 | / | / | / | / | / |
| *Rbmy1a1* | XX #3 | B6D2F1 | 0 | 1 | 11 | 0 | / | / | / | / | / |
| *Rbmy1a1* | XX #4 | B6D2F1 | 0 | 0 | 9 | 1 | / | / | / | / | / |
| *Rbmy1a1* | XX #5 | B6D2F1 | 0 | 3 | 18 | 4 | / | / | / | / | / |
| *Rbmy1a1* | XX #6 | B6D2F1 | 0 | 0 | 10 | 1 | / | / | / | / | / |
| *Rbmy1a1* | XX #7 | B6D2F1 | 0 | 1 | 9 | 0 | / | / | / | / | / |
| *Rbmy1a1* | XX #8 | B6D2F1 | 0 | 1 | 11 | 0 | / | / | / | / | / |
| *Ssty1* | XO #1 | C57BL/6 | 1 | 24 | 0 | 0 | Y | Y | Y | Y | Y |
| *Ssty1* | XO #2 | C57BL/6 | 2 | 13 | 10 | 0 | Y | Y | / | Y | Y |
| *Ssty1* | XO #3 | B6D2F1 | 3 | 14 | 0 | 0 | Y | Y | / | / | Y |
| *Ssty1* | XO #4 | B6D2F1 | 0 | 15 | 0 | 1 | Y | Y | / | / | Y |
| *Ssty1* | XO #5 | B6D2F1 | 1 | 24 | 0 | 0 | Y | / | / | Y | Y |
| *Ssty1* | XO #6 | B6D2F1 | 0 | 14 | 0 | 0 | Y | / | / | Y | Y |
| *Ssty1* | XO #7 | B6D2F1 | 0 | 15 | 0 | 0 | Y | / | / | Y | Y |
| *Ssty1* | XX #1 | C57BL/6 | 0 | 0 | 12 | 0 | / | / | / | / | / |
| *Ssty1* | XX #2 | C57BL/6 | 0 | 0 | 12 | 0 | / | / | / | / | / |
| *Ssty1* | XX #3 | C57BL/6 | 0 | 0 | 12 | 0 | / | / | / | / | / |
| *Ssty1* | XX #4 | C57BL/6 | 0 | 2 | 24 | 0 | / | / | / | / | / |
| *Ssty1* | XX #5 | C57BL/6 | 0 | 2 | 26 | 1 | / | / | / | Y | / |
| *Ssty1* | XX #6 | C57BL/6 | 1 | 2 | 12 | 1 | / | / | / | Y | / |
| *Ssty1* | XX #7 | B6D2F1 | 0 | 1 | 14 | 1 | / | / | / | / | / |
| *Ssty1* | XX #8 | B6D2F1 | 1 | 2 | 11 | 0 | / | / | / | / | / |
| *Ssty1* | XX #9 | B6D2F1 | 0 | 2 | 23 | 1 | / | / | / | / | / |
| *Ssty1* | XX #10 | B6D2F1 | 0 | 2 | 4 | 0 | / | / | / | / | / |
| *Ssty1* | XX #11 | B6D2F1 | 0 | 0 | 11 | 1 | / | / | / | / | / |
| *Ssty1* | XX #12 | B6D2F1 | 0 | 2 | 16 | 0 | / | / | / | Y | / |
| *Ssty1* | XX #13 | B6D2F1 | 0 | 2 | 9 | 0 | / | / | / | Y | / |
| *Ssty1* | XX #14 | B6D2F1 | 0 | 0 | 11 | 0 | / | Y | / | Y | / |
| *Ssty1* | XX #15 | B6D2F1 | 0 | 0 | 6 | 0 | / | / | / | Y | / |
| *Ssty1* | XX #16 | B6D2F1 | 0 | 1 | 12 | 0 | / | / | / | Y | / |
| *Ssty1* | XX #17 | B6D2F1 | 0 | 3 | 18 | 0 | / | Y | / | / | Y |
| *Ssty1* | XX #18 | B6D2F1 | 0 | 1 | 8 | 0 | / | / | / | / | / |
| *Ssty2* | XO #1 | C57BL/6 | 0 | 17 | 0 | 0 | Y | Y | Y | Y | Y |
| *Ssty2* | XO #2 | C57BL/6 | 1 | 17 | 0 | 0 | Y | / | / | Y | Y |
| *Ssty2* | XO #3 | C57BL/6 | 0 | 17 | 0 | 0 | Y | Y | Y | Y | Y |
| *Ssty2* | XO #4 | C57BL/6 | 0 | 14 | 0 | 0 | Y | Y | / | Y | Y |
| *Ssty2* | XO #5 | C57BL/6 | 1 | 10 | 0 | 0 | Y | Y | / | Y | Y |
| *Ssty2* | XO #6 | C57BL/6 | 1 | 20 | 3 | 0 | Y | Y | / | Y | Y |
| *Ssty2* | XO #7 | C57BL/6 | 3 | 27 | 0 | 0 | / | / | / | Y | / |
| *Ssty2* | XO #8 | B6D2F1 | 0 | 10 | 0 | 0 | Y | / | / | Y | Y |
| *Ssty2* | XO #9 | B6D2F1 | 2 | 11 | 0 | 0 | Y | / | / | Y | Y |
| *Ssty2* | XO #10 | C57BL/6 | 0 | 21 | 0 | 0 | / | / | / | Y | / |
| *Ssty2* | XO #11 | C57BL/6 | 0 | 8 | 0 | 0 | / | / | / | Y | / |
| *Ssty2* | XO #12 | C57BL/6 | 0 | 14 | 0 | 0 | / | / | / | Y | / |
| *Ssty2* | XO #13 | C57BL/6 | 0 | 23 | 0 | 0 | / | / | / | Y | / |
| *Ssty2* | XO #14 | C57BL/6 | 2 | 17 | 3 | 0 | / | / | / | Y | / |
| *Ssty2* | XO #15 | C57BL/6 | 1 | 11 | 0 | 0 | / | / | / | Y | / |
| *Ssty2* | XO #16 | B6D2F1 | 1 | 17 | 1 | 0 | / | / | / | Y | / |
| *Ssty2* | XO #17 | B6D2F1 | 1 | 17 | 0 | 0 | / | / | / | Y | / |
| *Ssty2* | XO #18 | B6D2F1 | 2 | 16 | 1 | 0 | / | / | / | Y | / |
| *Ssty2* | XO #19 | B6D2F1 | 1 | 17 | 2 | 0 | / | / | / | Y | / |
| *Ssty2* | XO #20 | B6D2F1 | 3 | 15 | 2 | 1 | / | / | / | Y | / |
| *Ssty2* | XO #21 | B6D2F1 | 0 | 20 | 0 | 0 | / | / | / | Y | / |
| *Ssty2* | XO #22 | B6D2F1 | 0 | 20 | 0 | 0 | / | / | / | Y | / |
| *Ssty2* | XO #23 | B6D2F1 | 0 | 20 | 0 | 0 | / | / | / | Y | / |
| *Ssty2* | XO #24 | B6D2F1 | 1 | 18 | 1 | 0 | / | / | / | Y | / |
| *Ssty2* | XO #25 | B6D2F1 | 1 | 18 | 1 | 0 | / | / | / | Y | / |
| *Ssty2* | XX #1 | C57BL/6 | 0 | 0 | 16 | 1 | / | / | / | / | / |
| *Ssty2* | XX #2 | C57BL/6 | 0 | 0 | 17 | 0 | / | / | / | / | / |
| *Ssty2* | XX #3 | C57BL/6 | 0 | 1 | 15 | 0 | / | / | / | / | / |
| *Ssty2* | XX #4 | C57BL/6 | 0 | 0 | 14 | 0 | / | / | / | / | / |
| *Ssty2* | XX #5 | C57BL/6 | 0 | 0 | 15 | 0 | / | / | / | / | / |
| *Ssty2* | XX #6 | C57BL/6 | 0 | 0 | 17 | 1 | / | / | / | / | / |
| *Ssty2* | XX #7 | C57BL/6 | 0 | 0 | 14 | 1 | / | / | / | / | / |
| *Ssty2* | XX #8 | C57BL/6 | 0 | 3 | 18 | 0 | Y | Y | / | Y | / |
| *Ssty2* | XX #9 | C57BL/6 | 0 | 1 | 13 | 1 | / | / | / | Y | / |
| *Ssty2* | XX #10 | C57BL/6 | 0 | 0 | 6 | 0 | / | / | / | Y | / |
| *Ssty2* | XX #11 | C57BL/6 | 1 | 0 | 11 | 0 | / | / | / | Y | / |
| *Ssty2* | XX #12 | C57BL/6 | 0 | 0 | 7 | 0 | / | / | / | Y | / |
| *Ssty2* | XX #13 | C57BL/6 | 0 | 1 | 16 | 0 | / | / | / | Y | / |
| *Ssty2* | XX #14 | C57BL/6 | 0 | 0 | 8 | 0 | / | / | / | Y | / |
| *Ssty2* | XX #15 | B6D2F1 | 0 | 2 | 13 | 0 | / | / | / | Y | Y |
| *Ssty2* | XX #16 | B6D2F1 | 0 | 0 | 15 | 0 | / | / | / | Y | / |
| *Ssty2* | XX #17 | B6D2F1 | 0 | 0 | 13 | 0 | / | / | / | Y | / |
| *Ssty2* | XX #18 | B6D2F1 | 1 | 1 | 7 | 0 | / | / | / | Y | / |
| *Ssty2* | XX #19 | C57BL/6 | 0 | 0 | 20 | 0 | / | / | / | / | / |
| *Ssty2* | XX #20 | C57BL/6 | 0 | 2 | 17 | 0 | / | / | / | / | / |
| *Ssty2* | XX #21 | C57BL/6 | 0 | 0 | 14 | 1 | / | / | / | / | / |
| *Ssty2* | XX #22 | C57BL/6 | 0 | 3 | 12 | 0 | / | / | / | / | / |
| *Ssty2* | XX #23 | C57BL/6 | 0 | 2 | 12 | 4 | / | / | / | / | / |
| *Ssty2* | XX #24 | C57BL/6 | 0 | 0 | 20 | 0 | / | / | / | / | / |
| *Ssty2* | XX #25 | C57BL/6 | 0 | 0 | 13 | 0 | / | / | / | / | / |
| *Ssty2* | XX #26 | C57BL/6 | 0 | 0 | 20 | 0 | / | / | / | / | / |
| *Ssty2* | XX #27 | C57BL/6 | 0 | 1 | 20 | 0 | / | / | / | / | / |
| *Ssty2* | XX #28 | C57BL/6 | 0 | 2 | 15 | 0 | / | / | / | / | / |
| *Ssty2* | XX #29 | C57BL/6 | 0 | 2 | 18 | 1 | / | / | / | Y | / |
| *Ssty2* | XX #30 | C57BL/6 | 0 | 0 | 27 | 0 | / | / | / | / | / |
| *Ssty2* | XX #31 | C57BL/6 | 0 | 0 | 22 | 0 | / | / | / | / | / |
| *Ssty2* | XX #32 | C57BL/6 | 0 | 1 | 19 | 1 | / | / | / | / | / |
| *Ssty2* | XX #33 | C57BL/6 | 0 | 0 | 18 | 0 | / | / | / | Y | / |
| *Ssty2* | XX #34 | C57BL/6 | 0 | 1 | 18 | 2 | / | / | / | Y | / |
| *Ssty2* | XX #35 | C57BL/6 | 0 | 0 | 21 | 0 | / | / | / | / | / |
| *Ssty2* | XX #36 | C57BL/6 | 0 | 0 | 19 | 0 | / | / | / | Y | / |
| *Ssty2* | XX #37 | C57BL/6 | 0 | 0 | 19 | 1 | / | / | / | / | / |
| *Ssty2* | XX #38 | B6D2F1 | 2 | 0 | 20 | 0 | / | / | / | Y | / |
| *Ssty2* | XX #39 | B6D2F1 | 0 | 0 | 16 | 2 | / | / | / | Y | / |
| *Ssty2* | XX #40 | B6D2F1 | 0 | 2 | 18 | 0 | / | / | / | Y | / |
| *Ssty2* | XX #41 | B6D2F1 | 1 | 1 | 20 | 0 | / | / | / | Y | / |
| *Ssty2* | XX #42 | B6D2F1 | 1 | 2 | 22 | 2 | / | / | / | / | / |
| *Ssty2* | XX #43 | B6D2F1 | 0 | 2 | 18 | 0 | / | / | / | / | / |
| *Ssty2* | XX #44 | B6D2F1 | 0 | 3 | 16 | 1 | / | / | / | / | / |
| *Ssty2* | XX #45 | B6D2F1 | 1 | 0 | 9 | 1 | / | / | / | / | / |
| *Ssty2* | XX #46 | B6D2F1 | 0 | 1 | 20 | 0 | / | / | / | / | / |
| *14-sgRNA* | XO #1 | B6D2F1 | 0 | 11 | 0 | 0 | Y | Y | / | / | / |
| *14-sgRNA* | XO #2 | B6D2F1 | 2 | 26 | 0 | 0 | Y | Y | / | / | / |
| *14-sgRNA* | XO #3 | B6D2F1 | 3 | 20 | 0 | 0 | Y | Y | / | / | / |
| *14-sgRNA* | XO #4 | B6D2F1 | 6 | 18 | 2 | 0 | Y | / | / | / | / |
| *14-sgRNA* | XO #5 | B6D2F1 | 1 | 8 | 14 | 0 | Y | / | / | / | / |
| *14-sgRNA* | XY/XO #1 | B6D2F1 | 0 | 15 | 5 | 0 | Y | Y | / | / | / |
| *14-sgRNA* | XY/XO #2 | B6D2F1 | 1 | 6 | 3 | 0 | Y | Y | / | / | / |
| *14-sgRNA* | XX #1 | B6D2F1 | 0 | 0 | 9 | 1 | / | / | / | / | / |
| *14-sgRNA* | XX #2 | B6D2F1 | 0 | 0 | 9 | 0 | / | / | / | / | / |
| *14-sgRNA* | XX #3 | B6D2F1 | 0 | 1 | 10 | 0 | / | / | / | / | / |
| *14-sgRNA* | XX #4 | B6D2F1 | 0 | 1 | 12 | 0 | / | / | / | / | / |
| *14-sgRNA* | XX #5 | B6D2F1 | 1 | 6 | 9 | 0 | Y | / | / | / | / |
| *14-sgRNA* | XX #6 | B6D2F1 | 0 | 2 | 8 | 0 | Y | / | / | / | / |
| *14-sgRNA* | XX #7 | B6D2F1 | 0 | 1 | 3 | 0 | / | / | / | / | / |
| *14-sgRNA* | XX #8 | B6D2F1 | 0 | 1 | 10 | 0 | / | / | / | / | / |
| *14-sgRNA* | XX #9 | B6D2F1 | 0 | 0 | 5 | 5 | / | / | / | / | / |
| *14-sgRNA* | XX #10 | B6D2F1 | 0 | 2 | 24 | 0 | / | / | / | / | / |
| *14-sgRNA* | XX #11 | B6D2F1 | 0 | 2 | 25 | 0 | / | / | / | / | / |
| *14-sgRNA* | XX #12 | B6D2F1 | 1 | 1 | 22 | 0 | / | / | / | / | / |
| *14-sgRNA* | XX #13 | B6D2F1 | 1 | 1 | 28 | 0 | / | / | / | / | / |
| *14-sgRNA* | XX #14 | B6D2F1 | 1 | 1 | 20 | 1 | / | / | / | / | / |
| *14-sgRNA* | XX #15 | B6D2F1 | 1 | 2 | 21 | 1 | / | / | / | / | / |
| *14-sgRNA* | XX #16 | B6D2F1 | 0 | 2 | 21 | 0 | / | / | / | / | / |
| *14-sgRNA* | XX #17 | B6D2F1 | 0 | 0 | 23 | 0 | / | / | / | / | / |
| *14-sgRNA* | XX #18 | B6D2F1 | 0 | 1 | 29 | 0 | / | / | / | / | / |
| *14-sgRNA* | XX #19 | B6D2F1 | 1 | 0 | 40 | 0 | / | / | / | / | / |
| *14-sgRNA* | XX #20 | B6D2F1 | 1 | 1 | 26 | 0 | / | / | / | / | / |
| *14-sgRNA* | XX #21 | B6D2F1 | 0 | 1 | 26 | 0 | / | / | / | / | / |
| *14-sgRNA* | XX #22 | B6D2F1 | 0 | 5 | 18 | 0 | / | / | / | / | / |
| *X-B* | XO #1 | B6D2F1 | 0 | 10 | 0 | 0 | / | / | Y | / | / |
| *X-B* | XO #2 | B6D2F1 | 0 | 23 | 1 | 0 | / | Y | / | / | / |
| *X-B* | XO #3 | B6D2F1 | 3 | 20 | 1 | 0 | / | Y | Y | / | / |
| *X-B* | XO #4 | B6D2F1 | 2 | 18 | 0 | 0 | / | Y | / | / | / |
| *X-B* | XO #5 | B6D2F1 | 3 | 20 | 0 | 0 | / | Y | / | / | / |
| *X-B* | XO #6 | B6D2F1 | 0 | 20 | 1 | 6 | / | / | / | / | / |
| *X-B* | XO #7 | B6D2F1 | 0 | 20 | 0 | 0 | / | / | / | / | / |
| *X-B* | XO/XX #1 | B6D2F1 | 0 | 11 | 11 | 0 | / | / | / | / | / |
| *X-B* | XX #1 | B6D2F1 | 0 | 1 | 14 | 0 | / | / | / | / | / |
| *X-B* | XX #2 | B6D2F1 | 0 | 1 | 15 | 2 | / | / | / | / | / |
| *X-B* | XX #3 | B6D2F1 | 2 | 2 | 16 | 0 | / | / | / | / | / |
| *X-B* | XX #4 | B6D2F1 | 0 | 3 | 17 | 0 | / | / | / | / | / |
| *X-B* | XX #5 | B6D2F1 | 0 | 2 | 17 | 0 | / | / | / | / | / |
| *X-B* | XX #6 | B6D2F1 | 0 | 0 | 19 | 0 | / | / | / | / | / |
| *X-B* | XX #7 | B6D2F1 | 1 | 1 | 17 | 0 | / | / | / | / | / |
| *X-B* | XX #8 | B6D2F1 | 1 | 2 | 17 | 0 | / | / | / | / | / |
| *X-B* | XX #9 | B6D2F1 | 0 | 0 | 19 | 0 | / | / | / | / | / |
| *X-B* | XX #10 | B6D2F1 | 0 | 1 | 20 | 1 | / | / | / | / | / |
| *X-B* | XX #11 | B6D2F1 | 0 | 0 | 14 | 1 | / | / | / | / | / |
| *X-B* | XX #12 | B6D2F1 | 0 | 0 | 19 | 0 | / | / | / | / | / |
| *X-B* | XX #13 | B6D2F1 | 0 | 2 | 18 | 1 | / | / | / | / | / |
| *X-C* | XO #1 | B6D2F1 | 1 | 7 | 2 | 0 | / | / | / | / | / |
| *X-C* | XO #2 | B6D2F1 | 3 | 9 | 3 | 0 | / | / | / | / | / |
| *X-C* | XO #3 | B6D2F1 | 1 | 8 | 2 | 0 | / | Y | / | / | / |
| *X-C* | XO #4 | B6D2F1 | 0 | 20 | 2 | 0 | / | Y | / | / | / |
| *X-C* | XO #5 | B6D2F1 | 1 | 19 | 1 | 0 | / | Y | Y | / | / |
| *X-C* | XO #6 | B6D2F1 | 1 | 13 | 3 | 0 | / | Y | / | / | / |
| *X-C* | XO #7 | B6D2F1 | 0 | 16 | 0 | 0 | / | Y | Y | / | / |
| *X-C* | XO #8 | B6D2F1 | 0 | 12 | 0 | 0 | / | / | / | / | / |
| *X-C* | XO #9 | B6D2F1 | 2 | 12 | 1 | 0 | / | / | / | / | / |
| *X-C* | XO #10 | B6D2F1 | 0 | 12 | 0 | 0 | / | / | / | / | / |
| *X-C* | XX #1 | B6D2F1 | 0 | 0 | 9 | 1 | / | / | / | / | / |
| *X-C* | XX #2 | B6D2F1 | 0 | 1 | 9 |  | / | / | / | / | / |
| *X-C* | XX #3 | B6D2F1 | 1 | 0 | 8 | 1 | / | / | / | / | / |
| *X-C* | XX #4 | B6D2F1 | 0 | 0 | 8 | 3 | / | / | / | / | / |
| *X-C* | XX #5 | B6D2F1 | 0 | 2 | 5 | 2 | / | / | / | / | / |
| *X-C* | XX #6 | B6D2F1 | 0 | 0 | 17 | 1 | / | / | / | / | / |
| *X-C* | XX #7 | B6D2F1 | 0 | 2 | 11 | 0 | / | / | / | / | / |
| *X-C* | XX #8 | B6D2F1 | 0 | 0 | 11 | 0 | / | / | / | / | / |
| *X-C* | XX #9 | B6D2F1 | 0 | 0 | 12 | 0 | / | / | / | / | / |
| *X-C* | XY #1 | B6D2F1 | 0 | 1 | 8 | 0 | / | / | / | / | / |
| *X-C* | XY #2 | B6D2F1 | 1 | 0 | 8 | 1 | / | / | / | / | / |
| *WT* | XX #1 | B6D2F1 | 0 | 2 | 16 | 0 | / | / | / | / | / |
| *WT* | XX #2 | B6D2F1 | 0 | 0 | 20 | 0 | / | / | / | / | / |
| *WT* | XX #3 | B6D2F1 | 0 | 1 | 15 | 5 | / | / | / | / | / |
| *WT* | XX #4 | B6D2F1 | / | / | / | / | / | Y | / | / | / |
| *WT* | XY #1 | B6D2F1 | 0 | 3 | 17 | 0 | / | / | / | / | / |
| *WT* | XY #2 | B6D2F1 | 0 | 1 | 19 | 0 | / | / | / | / | / |
| *WT* | XY #3 | B6D2F1 | 0 | 1 | 17 | 1 | / | / | / | / | / |
| *WT* | XY #4 | B6D2F1 | / | / | / | / | / | Y | / | / | / |
| *WT* |  | 129xC57BL/6 ESCs | 0 | 3 | 14 | 0 | / | Y | Y | / | / |
| *Rbmy1a1* | Mix | 129xC57BL/6 ESCs | 2 | 8 | 9 | 0 | / | Y | / | / | / |
| *Rbmy1a1* | Clone #2 | 129xC57BL/6 ESCs | 0 | 10 | 0 | 0 | Y | Y | Y | / | / |
| *Rbmy1a1* | Clone #3 | 129xC57BL/6 ESCs | 1 | 5 | 2 | 0 | / | / | / | / | / |
| *Rbmy1a1* | Clone #4 | 129xC57BL/6 ESCs | 3 | 4 | 3 | 0 | / | / | / | / | / |
| *Rbmy1a1* | Clone #5 | 129xC57BL/6 ESCs | 0 | 2 | 5 | 2 | / | / | / | / | / |
| *Rbmy1a1* | Clone #6 | 129xC57BL/6 ESCs | 2 | 5 | 2 | 0 | / | / | / | / | / |
| *Rbmy1a1* | Clone #7 | 129xC57BL/6 ESCs | 1 | 8 | 1 | 0 | / | / | / | / | / |
| *Rbmy1a1* | Clone #8 | 129xC57BL/6 ESCs | 0 | 8 | 2 | 0 | / | / | / | / | / |
| *Ssty2* | Mix | 129xC57BL/6 ESCs | 2 | 7 | 11 | 0 | / | Y | / | / | / |
| *Ssty2* | Clone #3 | 129xC57BL/6 ESCs | 1 | 16 | 1 | 0 | Y | Y | Y | / | / |
| *Ssty2* | Clone #4 | 129xC57BL/6 ESCs | 0 | 13 | 1 | 0 | / | / | / | / | / |
| *Ssty2* | Clone #5 | 129xC57BL/6 ESCs | 4 | 4 | 0 | 0 | / | / | / | / | / |
| *Ssty2* | Clone #6 | 129xC57BL/6 ESCs | 2 | 4 | 1 | 0 | / | / | / | / | / |
| *Ssty2* | Clone #7 | 129xC57BL/6 ESCs | 1 | 7 | 2 | 0 | / | / | / | / | / |
| *Ssty2* | Clone #8 | 129xC57BL/6 ESCs | 0 | 8 | 3 | 0 | / | / | / | / | / |
| *Ssty2* | Clone #9 | 129xC57BL/6 ESCs | 0 | 9 | 1 | 0 | / | / | / | / | / |
| *Ssty2* | Clone #10 | 129xC57BL/6 ESCs | 1 | 6 | 3 | 0 | / | / | / | / | / |
| *X-B* | mESCs #1 | Derive mESCs | 0 | 0 | 4 | 0 | / | / | / | / | / |
| *X-B* | mESCs #2 | Derive mESCs | 0 | 3 | 7 | 3 | / | / | / | / | / |
| *X-B* | mESCs #3 | Derive mESCs | 1 | 2 | 2 | 2 | / | / | / | / | / |
| *X-B* | mESCs #4 | Derive mESCs | 1 | 5 | 2 | 2 | / | / | / | / | / |
| *X-B* | mESCs #5 | Derive mESCs | 0 | 0 | 5 | 0 | / | / | / | / | / |
| *X-B* | mESCs #6 | Derive mESCs | 2 | 6 | 0 | 2 | / | / | / | / | / |
| *X-C* | mESCs #1 | Derive mESCs | 3 | 24 | 1 | 0 | / | Y | / | / | / |
| *X-C* | mESCs #2 | Derive mESCs | 7 | 21 | 1 | 0 | / | Y | / | / | / |
| *X-C* | mESCs #3 | Derive mESCs | 1 | 5 | 4 | 0 | / | / | / | / | / |
| *X-C* | mESCs #4 | Derive mESCs | 0 | 5 | 3 | 2 | / | / | / | / | / |

Note: Y, Experment performed; /, Not performed.

**Table S4.** Fertility of the female mice for chromosome elimination.

| Locus | Background | Name | Pregnancy | Parturition | Live pups |
| --- | --- | --- | --- | --- | --- |
| *Rbmy1a1* | C57BL/6J | XO/XY#1 | N | 0 | 0 |
|  |  | XO/XY#2 | N | 0 | 0 |
|  |  | XO/XY#3 | N | 0 | 0 |
|  |  | XO/XY#4 | N | 0 | 0 |
|  |  | XO#7 | N | 0 | 0 |
|  |  | XO#8 | N | 0 | 0 |
| *Ssty1* | C57BL/6J | XO#1 | Y | 0 | 0 |
|  |  | XO#2 | Y | 0 | 0 |
| *Ssty1* | B6DF1 | XO#5 | Y | 10 | 42 |
|  |  | XO#6 | Y |  |  |
|  |  | XO#7 | Y |  |  |
| *Ssty2* | C57BL/6J | XO#1 | N | 0 | 0 |
|  |  | XO#2 | Y | 1 | 0 |
|  |  | XO#3 | Y | 2 | 0 |
|  |  | XO#4 | N | 0 | 0 |
|  |  | XO#5 | Y | 2 | 0 |
|  |  | XO#6 | N | 0 | 0 |
|  |  | XO#7 | N | 0 | 0 |
|  |  | XO#13 | Y | 6 | 39 |
|  |  | XO#15 | Y |  |  |
|  |  | XO#10 | Y | 4 | 17 |
|  |  | XO#11 | Y |  |  |
|  |  | XO#12 | Y | 6 | 39 |
|  |  | XO#14 | Y |  |  |
| *Ssty2* | B6DF1 | XO#8 | Y | 3 | 19 |
|  |  | XO#9 | Y | 3 |  |
|  |  | XO#20 | Y | 1 | 0 |
|  |  | XO#24 | Y | 3 | 16 |
|  |  | XO#19 | N | 0 | 0 |
|  |  | XO#25 | Y | 1 | 5 |
|  |  | XO#16 | Y | 2 | 12 |
|  |  | XO#22 | Y | 1 | 1 |
|  |  | XO#17 | Y | 6 | 21 |
|  |  | XO#18 | Y |  |  |
|  |  | XO#21 | Y | 6 | 41 |
|  |  | XO#23 | Y |  |  |
| *Ssty1* | C57BL/6J | XX#5 | Y | 2 | 3 |
|  |  | XX#6 | Y | 2 | 0 |
| *Ssty1* | B6DF1 | XX#12 | Y | 15 | 73 |
|  |  | XX#13 | Y |  |  |
|  |  | XX#14 | Y |  |  |
|  |  | XX#15 | Y |  |  |
| *Ssty2* | C57BL/6J | XX#8 | Y | 2 | 8 |
|  |  | XX#9 | Y | 1 | 0 |
|  |  | XX#10 | Y | 1 | 7 |
|  |  | XX#11 | Y | 2 | 5 |
|  |  | XX#12 | Y | 4 | 9 |
|  |  | XX#13 | Y | 2 | 0 |
|  |  | XX#14 | Y | 3 | 21 |
|  |  | XX#29 | Y | 5 | 35 |
|  |  | XX#36 | Y |  |  |
|  |  | XX#33 | Y | 4 | 22 |
|  |  | XX#34 | Y |  |  |
| *Ssty2* | B6DF1 | XX#15 | Y | 2 | 71 |
|  |  | XX#16 | Y | 3 |  |
|  |  | XX#17 | Y | 2 |  |
|  |  | XX#18 | Y | 2 |  |
|  |  | XX#38 | Y | 7 | 50 |
|  |  | XX#40 | Y |  |  |
|  |  | XX#39 | Y | 6 | 56 |
|  |  | XX#41 | Y |  |  |

The 8-week-old female mice for chromosome elimination were paired with wild-type male mice for over three months.

**Table S6.** Overview of iHTGTS libraries.

| sgRNA | Total DNA for library | Final identified junctions |
| --- | --- | --- |
| NA | 20ug | 302 |
| *Kdm5d*-A | 20ug | 17130 |
| *Kdm5d*-A | 20ug | 14756 |
| *Kdm5d*-A | 20ug | 11413 |
| *Kdm5d*-A+*Ssty1*-A | 20ug | 3659 |
| *Kdm5d*-A+*Ssty1*-A | 20ug | 3461 |
| *Kdm5d*-A+*Ssty1*-A | 20ug | 3491 |
| *Kdm5d*-A+*Ssty1*-B | 20ug | 7697 |
| *Kdm5d*-A+*Ssty1*-B | 20ug | 7194 |
| *Kdm5d*-A+*Ssty1*-B | 20ug | 8715 |
| *Kdm5d*-A+*Ssty2*-A | 20ug | 4396 |
| *Kdm5d*-A+*Ssty2*-A | 20ug | 4067 |
| *Kdm5d*-A+*Ssty2*-A | 20ug | 5241 |
| *Kdm5d*-A+*Ssty2*-B | 20ug | 4732 |
| *Kdm5d*-A+*Ssty2*-B | 20ug | 5425 |
| *Kdm5d*-A+*Ssty2*-B | 20ug | 6589 |

**Table S7.** List of identified off-target sites by iHTGTS.

| Chromosome | Strand(+/-) | Start | End | Hotspot sequence | Frequency |
| --- | --- | --- | --- | --- | --- |
| ChrY | － | 916,845 | 916,867 | GATGGTACCTACAGAAGTTG TGG | On-target |
| ChrY | > 35 sites | | | CATCCCTCATGAAGAAGAGG AGG | On-target |
| ChrY | + | 2,003,920 | 2,004,428 | TATCCCTCAAGAAGAAGAGG AGG | 0.0398 |
| ChrY | – | 4,164,250 | 4,165,341 | CATCACTCAAGAAGAAGAGT AGG | 0.0428 |
| ChrY | – | 206,148 | 2,062,289 | CATCCCTCAAGAAGAAGAGG AGG | 0.0275 |
| ChrY | + | 1,581,542 | 1,582,134 | CATCCCTCAAGAAGAAGAGG AGG | 0.0248 |
| Chr18 | – | 85,716,199 | 85,716,862 | CATCCCTCAAGAAGAAGAGG AGG | 0.0180 |
| ChrY | + | 2,552,322 | 2,552,750 | CATCCCTCAAGAAGAAGAGG AGG | 0.0174 |
| ChrY | – | 2,380,967 | 2,381,629 | CATCCCTCAAGA-GAAAAGG AGG | 0.0164 |
| ChrY | > 35 sites | | | TGGAGCTCTACAGTGATGAC AGG | On-target |
| Chr5 | – | 125,848,278 | 125,848,616 | TGGAGCTCCGCAGTGATGAG AGG | 0.0164 |
| ChrY | + | 2,552,491 | 2,552,914 | AGAAGCTCCACAGAGATGAG AGG | 0.0151 |
| ChrY | > 30 sites | | | CATCACTCAAGAAGAAGAGT AGG | On-target |
| Chr18 | – | 85,716,200 | 85,716,789 | CATCCCTCAAGAAGAAGAGG AGG | 0.0062 |
| ChrY | + | 2,003,968 | 2,004,363 | TATCCCTCAAGAAGAAGAGG AGG | 0.0017 |
| ChrY | + | 2,552,324 | 2,552,719 | CATCCCTCAAGAAGAAGAGG AGG | 0.0018 |
| ChrY | – | 2,061,699 | 2,061,899 | CATCCCTCAAGAAGAAGAGG AGG | 0.0016 |
| ChrY | – | 2,337,167 | 2,337,401 | CATCCCTCAAGAAGAAGACG AGG | 0.0036 |
| ChrY | > 30 sites | | | AGGAGCTCCACAGCGATGAG AGG | On-target |
| ChrY | – | 4,164,415 | 4,164,563 | AGGAACTCCACGGCGATGAG AGG | 0.1496 |
| ChrY | – | 3,991,412 | 3,991,867 | AGGAGCTCCACAGTGATGAG AGG | 0.0248 |
| ChrY | – | 2,336,981 | 2,337,380 | AGGAGCTCCACAGCAATGAG AGA | 0.0140 |
| Chr18 | – | 85,716,192 | 85,716,701 | AGGAGCTTCACAGCGATGAG AGG | 0.0123 |

**Table S8.** Primers and oligos used in this work.

Oligo sequences for sgRNA construction used in chromosome elimination in culture cells.

| Chromosome | Locus | Sequence |
| --- | --- | --- |
|  |  |  |
| Mouse Y | *Rbmy1a1* | AF: CACCGTTCAAGTGATGATGGTCTCC |
|  |  | AR: AAACGGAGACCATCATCACTTGAAC |
|  |  | BF: CACCGTCCTTCATGTGAAGGGAACT |
|  |  | BR: AAACAGTTCCCTTCACATGAAGGAC |
|  | *Ssty2* | AF: CACCGCATCACTCAAGAAGAAGAGT |
|  |  | AR: AAACACTCTTCTTCTTGAGTGATGC |
|  |  | BF: CACCGAGGAGCTCCACAGCGATGAG |
|  |  | BR: AAACCTCATCGCTGTGGAGCTCCTC |
|  | *Kdm5d* | AF: CACCGATGGTACCTACAGAAGTTG |
|  |  | AR: AAACCAACTTCTGTAGGTACCATC |
|  |  | BF: CACCGGACTTATCTCCTGAAGAAA |
|  |  | BR: AAACTTTCTTCAGGAGATAAGTCC |
| Human Chr14 | Chr14-A | AF:CACCGAGCAACAACCTAGCATCTC |
|  |  | AR:AAACGAGATGCTAGGTTGTTGCTC |
|  | Chr14-B | BF:CACCGCGACTACGCGGACAGTGGTG |
|  |  | BR:AAACCACCACTGTCCGCGTAGTCGC |
|  | Chr14-C | CF:CACCGCTCGAAATCCTTGACAATCC |
|  |  | CR:AAACGGATTGTCAAGGATTTCGAGC |
|  | Chr14-D | DF:CACCGTTGCTCCTGAGGGTGCCAC |
|  |  | DR:AAACGTGGCACCCTCAGGAGCAAC |
|  | Chr14-E | EF:CACCGATAGGATGCGAGGTTTAGAT |
|  |  | ER:AAACATCTAAACCTCGCATCCTATC |
|  | Chr14-F | FF:CACCGAGAAGAGTTGGTTGGGGCGA |
|  |  | FR:AAACTCGCCCCAACCAACTCTTCTC |
| Human Chr21 | Chr21-A | AF:CACCGGAGGCTCGGTGCAGGTAAG |
|  |  | AR:AAACCTTACCTGCACCGAGCCTCC |
|  | Chr21-B | BF:CACCGCTGTGAGCATCCTCTGTGGA |
|  |  | BR:AAACTCCACAGAGGATGCTCACAGC |
|  | Control-A | AF:CACCGGCACGTACCGTGCCACACA |
|  |  | AR:AAACTGTGTGGCACGGTACGTGCC |
|  | Control-B | BF:CACCGCTCTGGGTCTCTAGAAGAA |
|  |  | BR:AAACTTCTTCTAGAGACCCAGAGC |
| Human Chr7 | HChr7-A | AF:CACCGACAGGTCTCCCGGGTGGCAC |
|  |  | AR:AAACGTGCCACCCGGGAGACCTGTC |
|  | HChr7-B | BF:CACCGAGTGATGTCATCATCCTGCG |
|  |  | BR:AAACCGCAGGATGATGACATCACTC |
|  | Control-A | AF:CACCGTGGGCAGATCACCTGAGGTC |
|  |  | AR:AAACGACCTCAGGTGATCTGCCCAC |
|  | Control-B | BF:CACCGCCTATAATCCCAGCTACAT |
|  |  | BR:AAACATGTAGCTGGGATTATAGGC |

Primers used for genotyping.

| Chromosome | Locus | Sequence | Product (bp) |
| --- | --- | --- | --- |
| Mouse Y (mESCs) | *Sry* | Outer F: GCACATTTTGGTCAGTGGCT | 632 |
|  |  | Outer R: GCTCTACTCCAGTCTTGCCT |  |
|  |  | Inner F: GTTCAGCCCTACAGCCACATG | 355 |
|  |  | Inner R: GCAGGCTGTAAAATGCCACTC |  |
|  | *Mecp2* | Outer F: ATAACTGGGCCAAACTGTGC | 436 |
|  |  | Outer R: GAGTCGCACATCTGTCTGGA |  |
|  |  | Inner F: TGCAGCTTCAGTTCACCTTG | 244 |
|  |  | Inner R: CCACTAACCACAGGCTCCAT |  |
| Mouse Y  (mouse tails) | *Uba1y* | F: CCTCATCACATCACCTCTTCTG | 814 |
|  |  | R: CTCACTTGCTCCAATCTTCACA |  |
|  | *Kdm5d* | F: CACTGAGAAGAAGATAGCCTAG | 898 |
|  |  | R: CTGAGAACCACTGATACATGAG |  |
|  | *Eif2s3y* | F: GCCTGTAAGATGTGGAAGATGAG | 801 |
|  |  | R: AGTAACTGACCTGCTATCCTCTG |  |
|  | *Ddx3y* | F: ACTCGTTACACTCGTCCTACTC | 1218 |
|  |  | R: CTTAGCCAGTCCAATCTCTATCAG |  |
|  | *Usp9y* | F: CAAGATAGCCTACATCAACCACAG | 806 |
|  |  | R: GAACCTACCACACTCATTCTACAC |  |
|  | *Tspy* | F: AGGTGAGTTGGAATTGTGTCTG | 919 |
|  |  | R: TGGAGAAGTGTGAAGTTGAAGG |  |
|  | *Rbmy1a1* | F: GCAGATGCTAAGAATGCTGTCA | 1785 |
|  |  | R: TACAACTCACACGCAATACTGG |  |
|  | *Ssty1* | F: GCCACTATAGCTGGATTATGAG | 1444 |
|  |  | R: GTCTTCACATCAGAGGTTCTAC |  |
|  | *Ssty2* | F: ACTCACTGTGTAGACCAGACTAG | 1677 |
|  |  | R: TCCAATTCCTTGCTCACTATGC |  |
|  | *Sry* | F: GCACATTTTGGTCAGTGGCT | 632 |
|  |  | R: GCTCTACTCCAGTCTTGCCT |  |
|  | *Gapdh* | F: TGAGTGGACCCTTCTTTGTAG | 585 |
|  |  | R: CATACCAGGAAATGAGCTTGAC |  |

Primers used for making template for in vitro transcription.

| Chromosome | Locus | Sequence |
| --- | --- | --- |
|  |  |  |
| Mouse Y | *Rbmy1a1* | A: TAATACGACTCACTATAGGGTTCAAGTGATGATGGTCTCCGTTTTAGAGCTAGAAATAG |
|  |  | B: TAATACGACTCACTATAGGGTCCTTCATGTGAAGGGAACTGTTTTAGAGCTAGAAATAG |
|  | *Ssty1* | A: TAATACGACTCACTATAGGGCATCCCTCATGAAGAAGAGGGTTTTAGAGCTAGAAATAG |
|  |  | B: TAATACGACTCACTATAGGGTGGAGCTCTACAGTGATGACGTTTTAGAGCTAGAAATAG |
|  | *Ssty2* | A: TAATACGACTCACTATAGGGCATCACTCAAGAAGAAGAGTGTTTTAGAGCTAGAAATAG |
|  |  | B: TAATACGACTCACTATAGGGAGGAGCTCCACAGCGATGAGGTTTTAGAGCTAGAAATAG |
|  | *Kdm5d* | A: TAATACGACTCACTATAGGGGATGGTACCTACAGAAGTTGGTTTTAGAGCTAGAAATAG |
|  |  | B: TAATACGACTCACTATAGGGGGACTTATCTCCTGAAGAAAGTTTTAGAGCTAGAAATAG |
|  | *Eif2s3y* | A: TAATACGACTCACTATAGGGACAATTGGTCATGTTGCTCAGTTTTAGAGCTAGAAATAG |
|  |  | B: TAATACGACTCACTATAGGGAGGAATATTACCATAAAACTGTTTTAGAGCTAGAAATAG |
|  | *Zfy1* | A: TAATACGACTCACTATAGGGGGATAGTGACCAGATTGTTGGTTTTAGAGCTAGAAATAG |
|  |  | B: TAATACGACTCACTATAGGGCCTGAGCAAGTTCTCAATTTGTTTTAGAGCTAGAAATAG |
|  | *Ube1y1* | A: TAATACGACTCACTATAGGGGAAACCCCTACTTGCCTCGCGTTTTAGAGCTAGAAATAG |
|  |  | B: TAATACGACTCACTATAGGGCTCAGCACAGCAGGCCTCCTGTTTTAGAGCTAGAAATAG |
|  | *Ddx3y* | A: TAATACGACTCACTATAGGGGTCTGTGATAAGGACAGTTCGTTTTAGAGCTAGAAATAG |
|  |  | B: TAATACGACTCACTATAGGGGTGATCGTGGAAGTGGATCCGTTTTAGAGCTAGAAATAG |
|  | *Usp9y* | A: TAATACGACTCACTATAGGGACTCTGGCTCTGTGTTTCCCGTTTTAGAGCTAGAAATAG |
|  |  | B: TAATACGACTCACTATAGGGAGGATTAGACTACCTTTGGAGTTTTAGAGCTAGAAATAG |
|  | *Zfy2* | A: TAATACGACTCACTATAGGGACTCTGGCTCTGTGTTTCCCGTTTTAGAGCTAGAAATAG |
|  |  | B: TAATACGACTCACTATAGGGAGGATTAGACTACCTTTGGAGTTTTAGAGCTAGAAATAG |
| Mouse X | X-A | TAATACGACTCACTATAGGGCTGGCTTCCTTACCCACTGCGTTTTAGAGCTAGAAATAG |
|  | X-B | TAATACGACTCACTATAGGGGCTTGGTTAGGGTGAGTGCTGTTTTAGAGCTAGAAATAG |
|  | X-C | TAATACGACTCACTATAGGGCTGGGAATTAGAATGCCAGAGTTTTAGAGCTAGAAATAG |
|  | X-D | TAATACGACTCACTATAGGGTAAGTGCTGTGTGCTGCTACGTTTTAGAGCTAGAAATAG |
|  | X-E | TAATACGACTCACTATAGGGTGTCATGACCTCTCTGTGACGTTTTAGAGCTAGAAATAG |
| Mouse Autosome | *Tyr*-A | TAATACGACTCACTATAGGGGGGAAGGGTTACTCAGAGTCGTTTTAGAGCTAGAAATAG |
|  | *Tyr*-B | TAATACGACTCACTATAGGGCCAGAAGCCAATGCACCTATGTTTTAGAGCTAGAAATAG |
|  | *Tyr*-C | TAATACGACTCACTATAGGGTACAGCTACCTCCAAGAGTCGTTTTAGAGCTAGAAATAG |
| Human Chr21 | HChr21-A | TAATACGACTCACTATAGGGGGAGGCTCGGTGCAGGTAAGGTTTTAGAGCTAGAAATAG |
|  | HChr21-B | TAATACGACTCACTATAGGGCTGTGAGCATCCTCTGTGGAGTTTTAGAGCTAGAAATAG |
|  | Control-A | TAATACGACTCACTATAGGGGGCACGTACCGTGCCACACAGTTTTAGAGCTAGAAATAG |
|  | Control-B | TAATACGACTCACTATAGGGGCTCTGGGTCTCTAGAAGAAGTTTTAGAGCTAGAAATAG |
| Universal reverse primer | sgRNA-R | AAAAGCACCGACTCGGTGCC |

| Cas9 mRNA | F | TAATACGACTCACTATAGGGAGATTTCAGGTTGGACCGGTG |
| --- | --- | --- |
|  | R | GACGTCAGCGTTCGAATTGC |

Primers used for off-target analysis.

| sgRNA | Locus | Sequence | Product (bp) |
| --- | --- | --- | --- |
| *Rbmy1a1*-A | *chr9:+106772566* | F: AAGGAGGAGGTGTTGGAGAC | 634 |
|  |  | R: GCATGAAGGCTAATAACTGTGG |  |
|  | *chr11:+12510761* | F: CTGCTTTGCTGACCAATCGTA | 666 |
|  |  | R: TGCTCCTCTTGACCTCATCTG |  |
|  | *chr8:-35350082* | F: GTGGAGGAATGTCACTATCTG | 909 |
|  |  | R: GACTATGTCCCAATCTACTGC |  |
|  | *chr2:-79819176* | F: CGTTAGAGGAGATGTGGTCAAT | 676 |
|  |  | R: GGAAGAAACTGTATGGCAAGAC |  |
|  | *chr18:-55264346* | F: CCCTATATGCTAAGCTCTGTAC | 641 |
|  |  | R: GATCCACTGATACACTGAGAC |  |
| *Rbmy1a1*-B | *chr7:-98841227* | F: GAAGATAAGATACGCCCACACT | 684 |
|  |  | R: AGACTGGACATGCCTATTCACT |  |
|  | *chr14:+20101586* | F: AGGCAACATGGTCAGATTCTTC | 928 |
|  |  | R: CCGAGGAGGAGTTCAATAATCAAG |  |
|  | *chr5:+20499940* | F: CGTTACCGCTCAATTCACAATG | 919 |
|  |  | R: CGTGGCAGTTCTTGATGGAAG |  |
|  | *chr3:-70119955* | F: ATCATATCCTTGCCACCTCTG | 692 |
|  |  | R: GATTGTCCTAGCACTCAGAAG |  |
|  | *chr5:+98508674* | F: GTTGGTTGTATCACTCTCCTTCAG | 934 |
|  |  | R: GCTCAGTAGAATTGTGGCATACC |  |
| *Ssty1-*A | *chr18:-85885975* | F: CGGAGGAGTCTCTGGAATGAT | 787 |
|  |  | R: TTGTGGTGCTAGGTGGTCTTA |  |
|  | *chr6:+34425091* | F: CTCCTGCCATTGCTAACTTCTG | 812 |
|  |  | R: GCTGTTCCACTTAGTGTCTTCTG |  |
|  | *chr10:+125133206* | F: ATGGTTGGCTGTGAGTATCTAC | 992 |
|  |  | R: GTCTTGGTCAAGGCATCTGTT |  |
|  | *chr15:+7934631* | F: CGAACACCGTAAGCAACAGAT | 631 |
|  |  | R: TGGCACTCACTTCAAGTCCTG |  |
|  | *chr9:+108619208* | F: GGAAGGTGGAAGACGGATGAA | 986 |
|  |  | R: GTGACTGCTGAACCTGACTGT |  |
| *Ssty1*-B | *chr11:+91809082* | F: TATGTGCTTACTGTGCTGAGAC | 609 |
|  |  | R: AGTTGCCTGCTTCTACTGAGA |  |
|  | *chr18:+38372217* | F: TTCGGCTCTTCACCTTACACT | 810 |
|  |  | R: TCATTCGTCTGACCTGGAACA |  |
|  | *chr13:-92843089* | F: CACAGATACCATCACTCAGTTC | 639 |
|  |  | R: CTGGGAGAAGCCATTCTACAA |  |
|  | *chr8:+33802701* | F: TCACAATGTTGACCCTGGAACC | 1007 |
|  |  | R: AGACTTCCCACTTGGAGTTCAG |  |
|  | *chr5:-134469634* | F: TGTCCTCCACTGATACCATCTAAG | 892 |
|  |  | R: GCAACCAGAGTCTGATTCCAAG |  |
| *Ssty2*-A | *chr18:-85885975* | F: GAAGGCTTGGCTAGAACTTGG | 827 |
|  |  | R: GACACATCTTGAGAAGGCAGAC |  |
|  | *chr13:-99256967* | F: CTTAGAAGGTTCCAGTCACAGT | 892 |
|  |  | R: GCCTCTGCTAAGAATGTTGATG |  |
|  | *chr2:+116264203* | F: AGCAGAGACCATAAGGAAGTGT | 745 |
|  |  | R: GAAGCACAGTTATCACCGATCT |  |
|  | *chr6:-76386849* | F: GGCAGAGTGGATAGCAATTACC | 722 |
|  |  | R: GGGAGTTTGTGTCAGAAAGAGG |  |
|  | *chr14:+47464580* | F: CTTCCTCACAGAGACAAGATG | 900 |
|  |  | R: CTCCTTCCTAGTAGCAGTTGA |  |
| *Ssty2-*B | *chr18:-85885780* | F: CAGAAGGCTTGGCTAGAACTTG | 829 |
|  |  | R: GACACATCTTGAGAAGGCAGAC |  |
|  | *chr17:-44626977* | F: AGGATGAAGGATGGTCTCTAGC | 821 |
|  |  | R: CAATCTGGTTACCTCTGCTTCC |  |
|  | *chr13:-50125357* | F: GACTTGACTGTGGAGCTGACA | 716 |
|  |  | R: GCTACCTCGCAATGAGACCTA |  |
|  | *chr2:-15832195* | F: GGAGGTTGGACTCAAGAACTG | 917 |
|  |  | R: ACACTACTTCCTGAGCCACAA |  |
|  | *chr9:+111245726* | F: GATGGAGGGCTAGAGAGATGG | 881 |
|  |  | R: GGTCAATGAGATGGCTTGCTAA |  |

Primers used for X chromosome damage identification.

| Locus | Sequence | Product (bp) |
| --- | --- | --- |
| X-C | F: CCATGAAGGTAACCAGTTCTC | 674 |
|  | R: CAGGGAGTGAAAGTATGAAAGG |  |
| *Gapdh* | F: TGAGTGGACCCTTCTTTGTAG | 585 |
|  | R: CATACCAGGAAATGAGCTTGAC |  |

1. Royo, H. et al. Evidence that Meiotic Sex Chromosome Inactivation Is Essential for Male Fertility. *Curr Biol* **20**, 2117-2123 (2010).
