# Supplementary material for: CRISPR/Cas9-mediated targeted chromosome elimination
Source: Genome Biol. 2017 Nov 24;18:224. doi: 10.1186/s13059-017-1354-4 (PMC5701507; doi:10.1186/s13059-017-1354-4)
Supplement: Additional file 4: Table S9. — Probes used in this work. (DOCX 509 kb) [file 13059_2017_1354_MOESM4_ESM.docx]

**Table S9.** Probes used in this work.

| Species | Name | Target | Dye Color | manufacturer | Catalog number | Use in figure |
| --- | --- | --- | --- | --- | --- | --- |
| Mouse | Chr X | XqA7.3 | Red | Guangzhou Exon Biotechnology | FD-5023M | 1, 2, 5, 6, 7, S4 |
|  | Chr Y | Whole Chr | Green | Empire Genomics | IDMF1057 | 1, 2,3, 5, S1 |
|  | Chr X | XqC3 | Red | Empire Genomics | MCEN-X-10-RE | 3, S1 |
|  | Chr 18 | 18qA1 | Green | Guangzhou Exon Biotechnology | FD-5318M | 8 |
| Human | Chr 14 | 14q11.2 | Green | Guangzhou Exon Biotechnology | FD-5114H | 7 |
|  | Chr 21 | Whole Chr | Red | Metasystem | D-0321-050-OR | 8 |
|  | Chr 21 | 21q22.11 | Green | Guangzhou Exon Biotechnology | FD-5021-H | 8 |
|  | Chr 7 | Whole Chr | Red | Metasystem | D-0307-050-OR | S4 |
